# Poisson-Gaussian Noise Reduction Using the Hidden Markov Model in Contourlet Domain for Fluorescence Microscopy Images

**DOI:** 10.1371/journal.pone.0136964

**Published:** 2015-09-09

**Authors:** Sejung Yang, Byung-Uk Lee

**Affiliations:** 1 Ewha Institute of Convergence Medicine, Ewha Womans University Medical Center, Seoul, Republic of Korea; 2 Department of Electronics Engineering, Ewha Womans University, Seoul, Republic of Korea; Beijing University of Technology, CHINA

## Abstract

In certain image acquisitions processes, like in fluorescence microscopy or astronomy, only a limited number of photons can be collected due to various physical constraints. The resulting images suffer from signal dependent noise, which can be modeled as a Poisson distribution, and a low signal-to-noise ratio. However, the majority of research on noise reduction algorithms focuses on signal independent Gaussian noise. In this paper, we model noise as a combination of Poisson and Gaussian probability distributions to construct a more accurate model and adopt the contourlet transform which provides a sparse representation of the directional components in images. We also apply hidden Markov models with a framework that neatly describes the spatial and interscale dependencies which are the properties of transformation coefficients of natural images. In this paper, an effective denoising algorithm for Poisson-Gaussian noise is proposed using the contourlet transform, hidden Markov models and noise estimation in the transform domain. We supplement the algorithm by cycle spinning and Wiener filtering for further improvements. We finally show experimental results with simulations and fluorescence microscopy images which demonstrate the improved performance of the proposed approach.

## Introduction

Digital images are prevalent in our lives as a result of advances in multimedia, internet, computers, and wide spread of portable imaging devices such as consumer digital cameras and camcorders. Image acquisition and processing is also common in medical technology and the service industry. Such demands have consequently led to the advancement of image sensors such as the charge coupled device (CCD) and the complementary metal oxide semiconductor (CMOS). Owing to the development of image sensor hardware, increased spatial resolution has resulted in a decreased sensor pixel size, which causes the expansion of the photon noise effect [1]. In many image applications such as fluorescence microscopy and astronomy, only a limited number of photons can be collected due to various physical constraints such as a light source with low power to avoid phototoxicity, and short exposure time. Therefore, these applications acquiring images by photon counting have extremely low signal to noise ratio [2]. The aim of this study is to model and remove the noise in a low-count image.

The two predominant sources of noise in digital image acquisition are (a) the stochastic nature of the photon-counting process at detectors, and (b) the intrinsic thermal and electronic fluctuations of the acquisition devices. Traditional denoising algorithms have employed the additive white Gaussian noise modeling to account for the second source of noise, which is signal-independent. Robbins proposed an empirical Bayesian framework to estimate Gaussian noise [3]. Lee, likewise, proposed a two-step empirical Bayesian estimation [4] which estimates the variance of signal from the neighbors of an observed pixel and applies the standard linear least squares (LLS) solution. Malfait and Roose further exploited a methodology to realize the Bayesian approach by applying the Markov random field [5] and Crouse *et al*. proposed an algorithm which uses hidden Markov models (HMM) to obtain the variance of signal and therefore, to denoise with Bayesian estimation [6]. Simoncelli published a research paper on the Bayesian denoising process [7], and Mihcak *et al*. applied a maximum a posteriori (MAP) estimation based on exponential marginal prior [8]. The Bayesian least squares-Gaussian scale mixtures (BLS-GSM) algorithm developed by Portilla *et al*. consists of a multivariate estimator resulting from Bayesian least-squares optimization, assuming Gaussian scale mixtures as a prior for neighborhoods of coefficients at adjacent positions and scales [9]. Block Matching 3D (BM3D), regarded as one of the state-of-the-art methods, improves sparsity by grouping 2D image blocks into 3D data sequences and decreases noise effectively by collaborative Wiener filtering [10]. These algorithms show reasonable performance for signal-independent Gaussian noise. However, the reduction in the size of an image sensor has resulted in a higher impact of signal-dependent noise. Consequently, the mixed Gaussian-Poisson noise model was developed [1]. Since Poisson noise is signal-dependent, it cannot have a constant noise variance, which causes difficulties in designing a denoising algorithm. To overcome the difficulties, variance stabilizing transforms (VSTs) such as the Anscombe transform [11] and the Fisz transform [12] have been introduced. Donoho exploited the Anscombe VST in denoising applications [13]. Later, many noise removal techniques employed the VST with the denoising algorithms based on the Gaussian noise reduction algorithm for Poisson noise removal [14–19]. Although the Anscombe VST makes the variance of Poisson noise constant and allows the denoising algorithms based on Gaussian noise to perform effectively, it shows disappointing results in the case of low-count images [17–19]. This is because of the inverse transformation rather than the stabilization itself. Bias error is unavoidable after an inverse transformation since the Anscombe transform is a nonlinear transformation. To overcome this bias problem, Mäkitalo and Foi proposed the exact unbiased inverse of the generalized Anscombe transform (GAT) for Poisson-Gaussian noise [20–22]. In this paper, we tested the combination of GAT and the existing noise reduction method based on Gaussian noise for comparison with the proposed algorithm.

Another effective method for Poisson noise reduction is an algorithm using the Haar transform. Poisson data after the Haar transform have a binomial distribution for children coefficients given a parent coefficient. This property has been exploited not only in user-calibrated hypothesis testing [23] but in the Bayesian framework as well [18] [24–26]. Since the Gaussian noise reduction methods yield higher quality images with the differentiable wavelet transform than with the Haar transform or other shift-invariant transformations, researchers have begun to modify Poisson-intensity estimation in order to incorporate the multi-scale transformation. Kolaczyk developed a shrinkage method using corrected hard/soft threshold based on an arbitrary wavelet transform to handle the nature of burst-like Poisson intensities [27], and Charles and Rasson generalized this approach to operate for various kinds of Poisson data [28]. Nowak and Baraniuk proposed a wavelet shrinkage method where the threshold is locally estimated based on cross validation estimation [29]. Lingenfelter *et al*. presented the optimal penalty function to produce sparser images for maximum-likelihood solution with Poisson data [30]. Recently, a sparsity-regularized convex optimization algorithm for Poisson noise has been developed [31], and Salmon *et al*. improved this approach using non-local Principal Component Analysis for Poisson noise [32]. Giryes and Elad proposed a Poisson denoising method using sparse representation modeling by Salmon’s method and dictionary learning [33].

Although the Poisson noise removal framework has been exploited in many publications as stated above, there has been very little research on mixed Poisson-Gaussian noise removal. Boulanger *et al*. proposed an effective denoising method of 3D fluorescence microscopy images for Poisson-Gaussian noise [34]. This method employs the generalized Anscombe transform to stabilize the variance of Poisson-Gaussian noise and achieves noise reduction using a minimizer consisting of an objective non-local energy functional involving spatio-temporal image patches. Poisson-Gaussian unbiased risk estimate-linear expansion of thresholds (PURE-LET) by Luisier *et al*. is a recent study on this issue [2][19][35]. This methodology optimizes a thresholding algorithm in the transform domain of the undecimated wavelet transform (UWT) and block discrete cosine transform (BDCT) to denoise images corrupted by mixed Poisson-Gaussian noise. PURE-LET minimizes the noise according to the data-adaptive unbiased estimation of the mean squared error (MSE) by a non-Bayesian framework. This algorithm plays an important role in treating Poisson-Gaussian noise directly. However, PURE-LET approximates the unbiased MSE estimation to minimize the cost function. In this paper, we propose an effective noise removal algorithm for Poisson-Gaussian noise using HMM and contourlet transform. The Poisson-Gaussian distribution model is used to estimate the noise parameters of an image, and these parameters are applied to our proposed denoising algorithm for noise reduction.

## Materials and Methods

### Fluorescence Microscopy image data set

The first set of images was acquired from a Nikon C1 Plus confocal laser microscope at the Medicinal Bioconvergence Research Center at Seoul National University. The data set contained 100 samples with a 512x512 size of fixed HeLa cells, labeled with three fluorescent dyes: Alexafluor555 in the red channel, Alexafluor488 in the green channel, and DAPI in the blue channel. The average of 100 images was used as the baseline for PSNR calculation.

The second data set was obtained from a Nikon A1R confocal laser microscope at the Department of Life Science of Ewha W. University. The data set contained 40 images of fixed HeLa cells of 512x512 size, labeled with two fluorescent dyes: golgin97 in the green channel, and DAPI in the blue channel. The average of 40 images was used as the baseline also.

### Noise modeling and estimation

Noise can be classified into signal-dependent noise and signal-independent noise. Signal-dependent noise is modeled by a Poisson distribution which is obtained from photon counting and signal-independent noise is normally modeled by a Gaussian distribution [36].

In this section, we follow the Poisson-Gaussian noise modeling of Foi *et al*. [1]. The observed signal *z* can be represented as the sum of the original signal *y* and noise as

$$z(x)=y(x)+\eta_{p}\left(y(x)\right)+\eta_{g}(x)\;,$$ (1)

where *x* ∈ *X* is the pixel position in the image domain *X*. The noise model is represented with two mutually independent parts, the signal-dependent Poisson component *η*_*p*_, and the signal-independent Gaussian component *η*_*g*_.

Assume that *χ*(*y*(*x*) + *η*_*p*_(*y*(*x*))) has the Poisson distribution, where *χ* > 0 is a scale factor. The probability distributions of Poisson and Gaussian noise are denoted as follows:

$$\chi \left(y(x)+\eta_{p}\left(y(x)\right)\right)∼P\left(\chi y(x)\right),\;\text{and}\;\eta_{g}(x)∼N(0,b),$$ (2)

where *χ* > 0 and *b* ≥ 0 are real scalar parameters and *P*(∙) and *N*(∙) represent the Poisson and normal distributions. The mean and the variance of the Poisson distribution are the same. Therefore, the variance can be obtained from the intrinsic property of a Poisson distribution as follows:

$$\text{var}\left\{\chi \left(y(x)+\eta_{p}\left(y(x)\right)\right)\right\}=E\left\{\chi \left(y(x)+\eta_{p}\left(y(x)\right)\right)\right\}=\chi y(x).$$ (3)

Since *E*{*η*_*p*_ (*y*(*x*))} = 0 and *χ* ^2^ var{*η*_*p*_ (*y*(*x*))} = *χy*(*x*), the variance can be represented as var{*η*_*p*_ (*y*(*x*))} = *y*(*x*) / *χ* from Eq (3). Therefore, the Poisson noise component *η*_*p*_ has a variance proportional to the signal *y*(*x*). That is var{*η*_*p*_ (*y*(*x*))} = *ay*(*x*), where *a* = *χ* ^−1^. On the other hand, the variance of Gaussian noise *η*_*g*_ is constant and it will be represented as *b*.

As a result, the overall noise variance of *z* in Eq (1) has an affine form,

$$\sigma^{2}\left(y(x)\right)=ay(x)+b.$$ (4)

The standard deviation *σ* becomes $\sigma \left(y(x)\right)=\sqrt{ay(x)+b}$. In this paper, the minimum and the maximum value of brightness *y*(*x*) are normalized to 0 and 1, respectively. Thus the standard deviation ranges from $\sigma (0)=\sqrt{b}$ to $\sigma (1)=\sqrt{a+b}$.

The Poisson noise parameters are estimated using the noise estimation algorithm in [1][37]. The noise parameter estimation process is as follows. Firstly, the image in the wavelet domain is segmented into smooth regions using level sets. Secondly, local mean and variance are estimated for each uniform region after the wavelet analysis. Finally, the optimal parameters $\hat{a}$ and $\hat{b}$ are estimated by maximum likelihood (ML) estimation.

$$(\hat{a},\hat{b})=\underset{a,b}{\text{arg max}}\;L(a,b)=\underset{a,b}{\text{arg min}}\;\left(-\text{ln}\;L(a,b)\right),$$ (5)

where $L(a,b)=\prod_{i=1}^{N}\int_{-\infty }^{\infty }p(({\hat{y}}_{i},{\hat{\sigma }}_{i})|y_{i}=y)p_{0}(y)dy$. Eq (5) can be rewritten as

$$(\hat{a},\hat{b})=\underset{a,b}{\text{arg min}}\;\left(-\sum_{i=1}^{N}\text{ln}\;\int p(({\hat{y}}_{i},{\hat{\sigma }}_{i})|y_{i}=y)p_{0}(y)dy\right).$$ (6)

The noise variance of *z* is therefore calculated using Eq (4) and the estimated parameters $\hat{a}$ and $\hat{b}$.

### Contourlet Transform

Signal modeling in the wavelet domain has been employed for an effective image noise reduction. Most natural images, however, are composed of not only discontinuous corners but also smooth curves. While the wavelet can express corners, it cannot capture the smoothness along contours in a compact manner. Hence, new algorithms such as curvelet and contourlet transforms have been developed to improve such shortcomings [38–39].

The curvelet transform, which was developed for better noise removal, consists of a 2-D rotation operation and a frequency domain division based on the polar coordinates [38]. As a result, information of the various directional features in images can be obtained to retain curves and edges compactly. The decomposition into the curvelet is simple in the continuous domain, but becomes problematic in the discrete domain. Geometrical problems such as significant bias in horizontal and vertical directions appear in the generalized rectangular-sampling grid. To overcome such difficulties, Do and Vetterli proposed the contourlet transform, which has similar effectiveness as the curvelet transform, for the direct use in the discrete domain [39].

**Fig 1:**
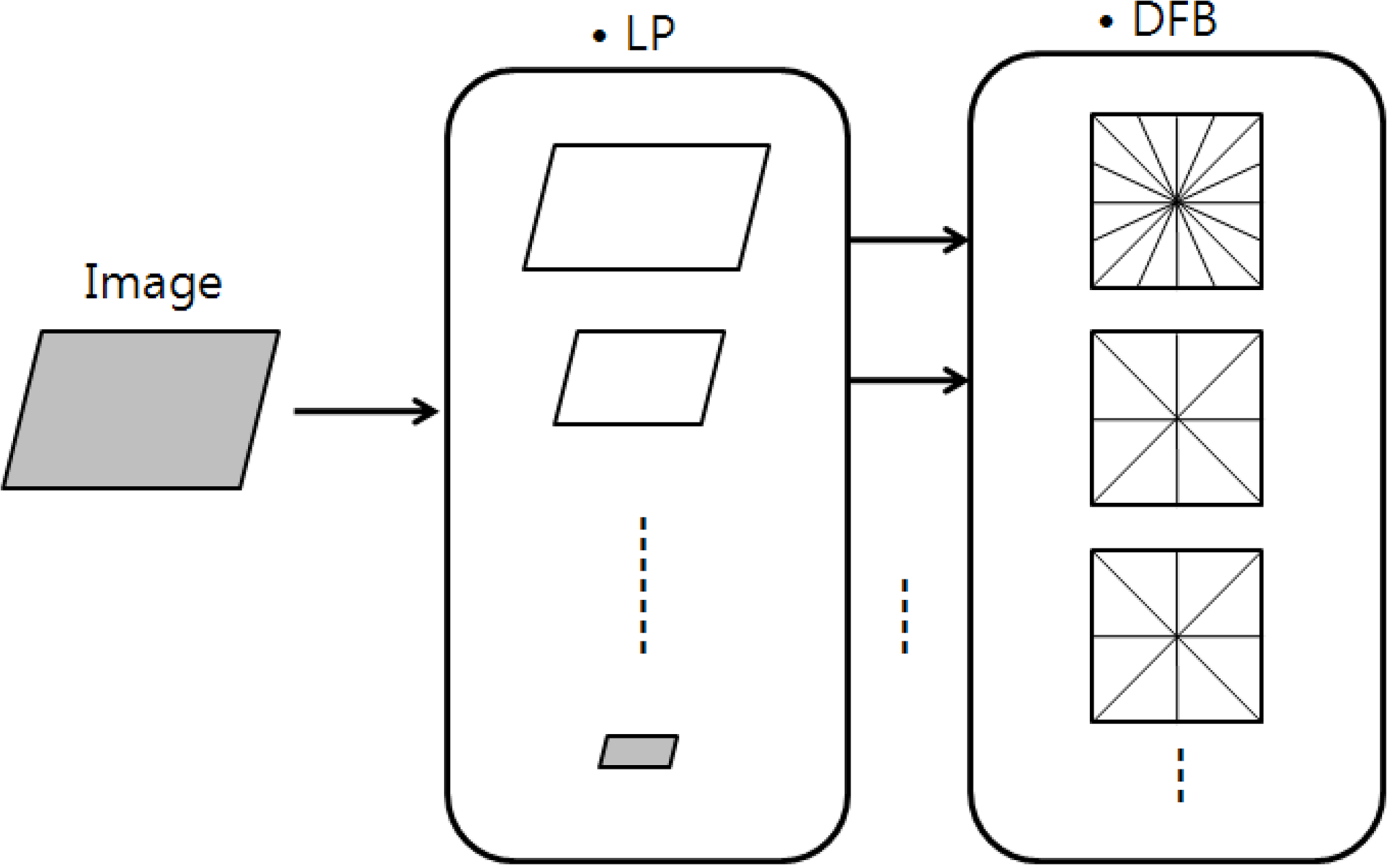
Contourlet Filter bank consisting of Laplacian Pyramid (LP) and Directional Filter Bank (DFB) [39].

The contourlet transform consists of two filters: the Laplacian pyramid (LP) filter and the directional filter bank (DFB). The LP filter is used in the existing wavelet filter as a low pass filter to separate high and low frequencies. The directional filter bank provides information on image direction components as described in Fig 1. After passing the LP filter, images are partitioned into high and low frequencies. While the high frequency component is divided according to direction in the DFB, the low frequency component is, after down sampling, divided into low and high frequency bands by the LP filter. DFB filtering is recursively applied to high frequency components. Since the contourlet transform can extract multi-resolution and multi-directional information, the boundaries of natural images can be described effectively. Typically, the LP filter proposed by Burt and Adelson is used [40]. The LP filter produces band-pass images by generating image differences between the down-sampled images and the original images. Although the biggest drawback of the LP filter is over sampling, the filter produces desired band-pass images without mixing frequency components at each pyramid level. While the wavelet filter can mix frequencies in the high-band channel after down sampling, the LP filter does not mix frequencies since the images that only pass the low frequency filter are downsampled [41]. In many cases, the DFB filter is based on the design of Bamberger and Smith [42]. The directional filter produces wedge-shaped frequency division subbands for 2^ℓ^ at ℓ—level binary tree decomposition.

**Fig 2:**
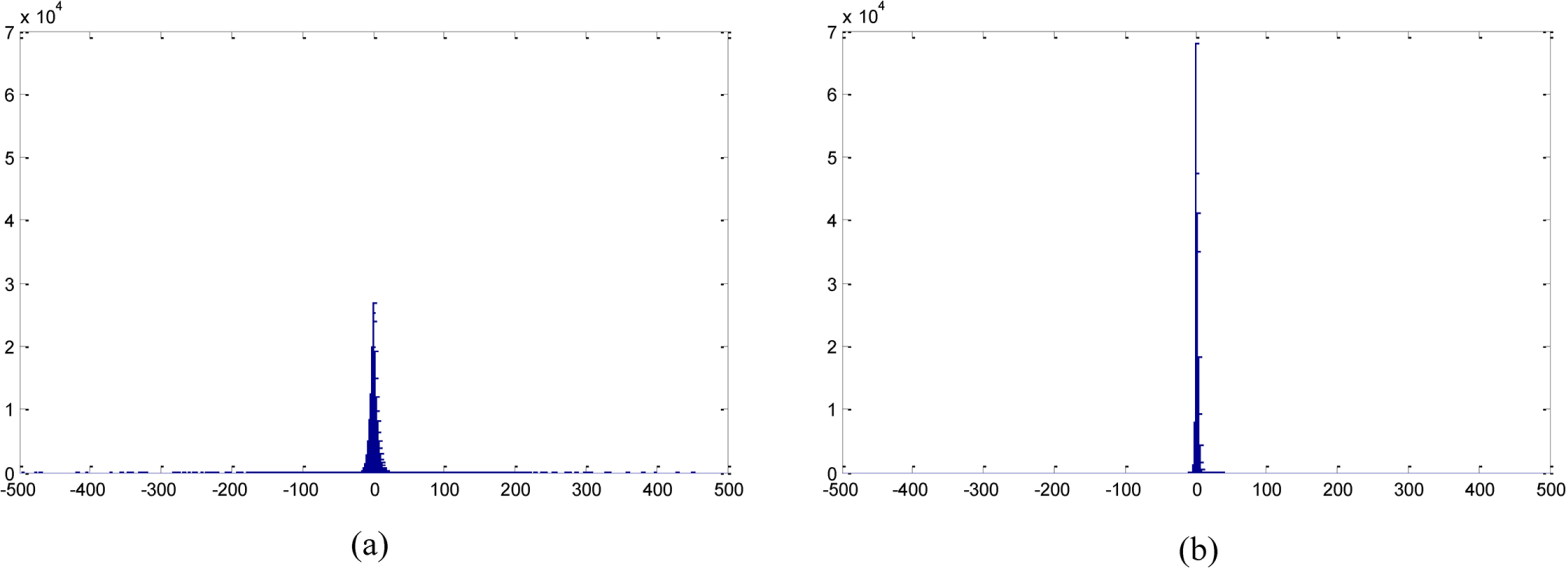
The comparison of sparsity between wavelet and contourlet transforms on the *Lena* image. (a) wavelet, (b) contourlet.

To evaluate the sparsity between the wavelet transform and the contourlet transform, the histograms of their coefficients are compared with respect to the *Lena* image after adding Poisson-Gaussian noise (S1 Fig). The wavelet filter and directional filter used for the comparison are ‘Daubeichies8’ and ‘dmaxflat5’, respectively. The filter type does not practically affect the performance. Fig 2 shows that the sparsity of the contourlet transform is much higher than that of the wavelet transform. The ratio of zeros in the contourlet coefficients is 4.83%, while that in the wavelet coefficients is 1.88%. Zeros in the contourlet coefficients is almost twice as many as that in the wavelet coefficients.

The contourlet filter bank achieves continuous domain expansion at $L_{2}({ℜ}^{2})$ using the contourlet transform. The connection between discrete contourlet transform and continuous domain expansion can be established with a new multiresolution analysis structure in a similar method of the link between the wavelet transform and filterbank [43].

### The Contourlet HMM for Poisson-Gaussian Noise Reduction

Signal modeling, as well as the noise pdf, is important for effective noise removal. Existing algorithms have applied a generalized Gaussian [25] or Gaussian scale mixtures [6] for signal modeling. In this paper, the HMM method proposed by Crouse *et al*. is adopted to model the signal pdf for its efficacy [3]. In this section we summarize the HMM signal modeling and present our extension for Poisson-Gaussian noise.

**Fig 3:**
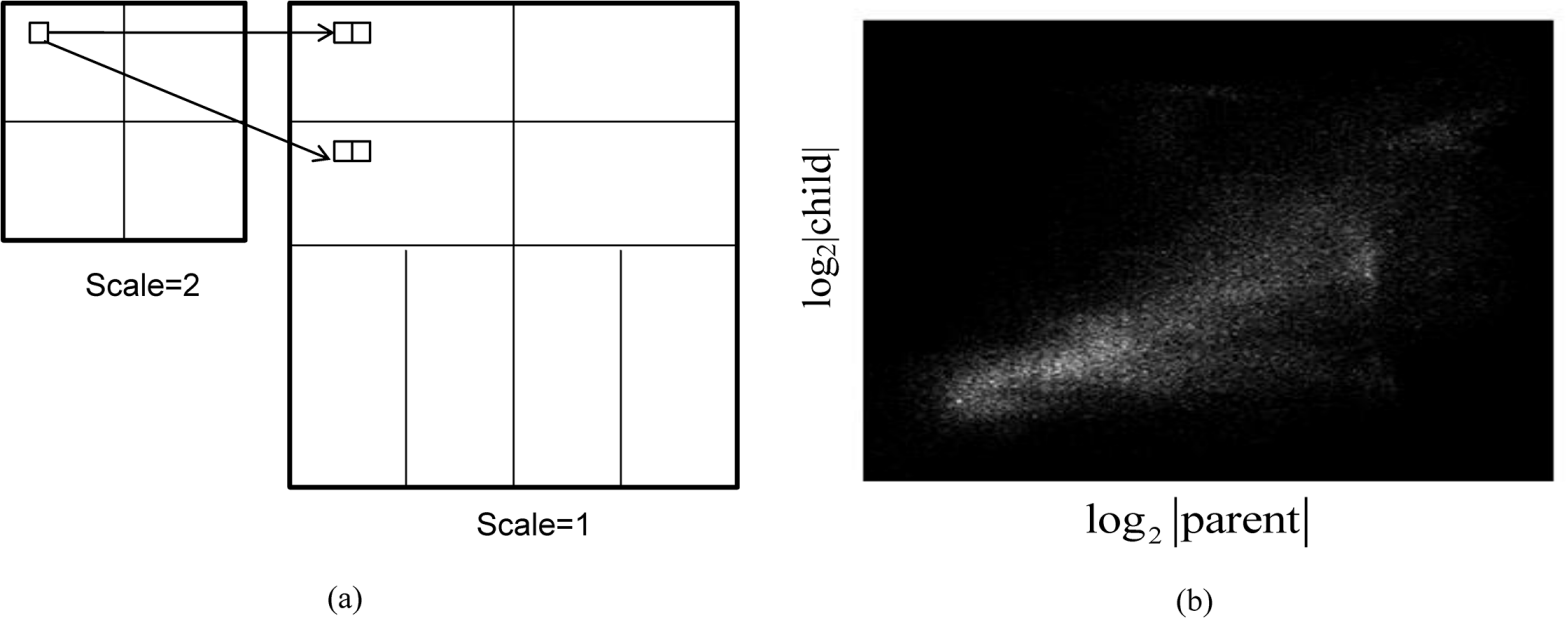
Parent-child relationship of contourlet coefficients. **(**a) a parent coefficient and its pertinent four child coefficients (b) parent-child dependency of contourlet coefficients in log scale.

The HMM denoising method by Crouse *et al*. is based on the wavelet transform and the signal-independent noise model. In this paper, the contourlet transform is employed, whereas the Crouse’s method uses the wavelet transform. The contourlet transform results in better sparsity than the wavelet transform as shown in Fig 2. The marginal distribution of natural images in contourlet domain is highly non-Gaussian. Furthermore, the dependency relationship exists between the parent and child coefficients in the contourlet transform, which is an important reason for applying the HMM. As shown in Fig 3(A), the parent coefficient has its four children in two separate directional sub-bands, while parent-children relationship in the wavelet domain is always in the same direction. Fig 3(B) plots the absolute values of the parent and child coefficients in log scale. It demonstrates the dependency between the parent and the child coefficients of the *Lena* image.

The contourlet HMM utilizes the clustering and persistence properties of contourlet transform coefficients. Clustering and persistence are additional useful properties of contourlet transform besides major properties such as locality, multiresolution and energy compaction. Clustering is the property where adjacent coefficients tend to be large (small), if a contourlet coefficient value is large (small). Persistence shows that the values of contourlet coefficients are very likely to propagate across scales; if a contourlet coefficient is large (small) in one scale, then the parent coefficient is large (small).

Two statistical models can describe contourlet properties in HMM. First, the independent mixture model is used to decompose the marginal probability of each coefficient as a mixture density with a hidden state variable to reflect the non-Gaussian property of the contourlet coefficients. Second, probabilistic graphs are used to represent the dependencies between contourlet coefficients. The hidden Markov chain model captures the horizontal dependencies within each scale and the hidden Markov tree (HMT) represents the vertical dependencies across scale.

The mixture model is composed of two-state zero-mean gaussians: the pdfs of the state variable *S*, *p*_*S*_(1) and *p*_*S*_(2) = 1 − *p*_*S*_(1), and the variances of Gaussian pdfs in each state. In most of the mixture model applications, the contourlet coefficient *W* is observable, however, the state variable *S* cannot be observed, thus appropriately called *hidden*.

In general, an *M*-state Gaussian mixture model of random variable *W* is given as follows.

1. a discrete random state variable *S* which has the values *s* ∈ 1, 2, ⋯, *M*, according to the pdf *p*_*S*_(*s*).
2. the Gaussian conditional pdfs *p*_*W*|*S*_ (*w* | *S* = *s*), *s* ∈ 1, 2, ⋯, *M*.

Hence, the probability density function *p*_*W*_ is a weighted sum of the Gaussian conditional pdfs:

$$p_{W}(w)=\sum_{m=1}^{M}p_{S}(m)p_{W|S}(w|S=m).$$ (7)

The HMM parameters for the *M*-state Gaussian mixture model for each contourlet coefficient *W*_*i*_ are

1. $p_{S_{1}}(m)$: the pdf for the root node *S*_1_
2. $\varepsilon_{i,\rho (i)}^{mr}=p_{S_{i}|S_{\rho (i)}}[m|S_{\rho (i)}=r]$: the conditional probability that *S*_*i*_ is in state *m* when the given *S*_*ρ*(*i*)_ is in state *r*
3. *μ*_*i*,*m*_ and $\sigma_{i,m}^{2}$: the mean and variance of contourlet variable *W*_*i*_ when the given *S*_*i*_ is in state *m*.

These parameters can be grouped by model parameter vector **θ.**

To estimate the HMM parameters for given data, the expectation maximization (EM) algorithm is employed. The training data *w* is incomplete; the complete data (**w**, **s**) is composed of the training data and the hidden state **s**. The goal is then to maximize the incomplete log-likelihood function ln *p*(**w** | θ), where the EM algorithm performs this challenging maximization with an iterative process separately taking two simple steps, E step and M step. The E step calculates the expected value *E*_*S*_[ln *p*(**w**, **S** | θ) | **w**, θ^*l*^] in the *l* th iteration. Then the M step maximizes the log-likelihood function of θ to acquire θ^*l*+1^ for the next iterative process. Once the parameters are obtained, then the Bayesian noise removal process can be applied. After the contourlet coefficient $w_{i}^{k}$ of the noise signal and the state $s_{i}^{k}$ are obtained, the conditional estimation for the contourlet coefficient $v_{i}^{k}$ of the original signal is as follows:

$$E[V_{i}^{k}|W_{i}^{k}=w_{i}^{k},\;S_{i}^{k}=m]=\frac{\sigma_{i,m}^{2}}{\sigma_{n_{i}}^{2}+\sigma_{i,m}^{2}}w_{i}^{k}\;,$$ (8)

where $\sigma_{n_{i}}^{2}$ is the noise variance in contourlet domain.

The conditional mean estimation of $v_{i}^{k}$ can be obtained by using the hidden state probabilities $p(S_{i}^{k}|w^{k},\;\theta )$ as by-products of the EM algorithm through the chain rule of conditional expectation.

$$E[v_{i}^{k}|w^{k},\;\theta ]=\sum_{m}p(S_{i}^{k}=m|w^{k},\;\theta )\times \frac{\sigma_{i,m}^{2}}{\sigma_{n_{i}}^{2}+\sigma_{i,m}^{2}}w_{i}^{k}$$ (9)

Finally the denoised signal is acquired by the inverse transform of the estimated contourlet coefficient.

In what follows, we describe the denoising method for Poisson-Gaussian noise. While the variance of Gaussian noise is constant, the variance of Poisson-Gaussian noise is related to the signal intensity. In other words, each pixel has different noise variance and thus it cannot be controlled using the traditional HMM. Therefore, we extend the contourlet HMM to deal with Poisson-Gaussian noise (PG-HMM). The parameters of Poisson-Gaussian noise *a* and *b* defined in Section 2, which are the noise estimates in the image domain, can be estimated by the noise estimation method in [1]. The variance of the noise component in the image domain in Eq (1) is denoted as $\sigma_{e_{i}}^{2}$, where *e*(*x*) = *η*_*p*_ (*y*(*x*)) + *η*_*g*_ (*x*) denotes the sum of Poisson and Gaussian noise which can be calculated by Eq (4). The original signal *y*_*i*_ is approximated by the low-pass filtered value of the noisy image. We then need to estimate the noise variance in contourlet domain. Let *z*_*i*_ and *h*_*i*_ denote the observed pixel values and the contourlet filter coefficients, respectively. The noise component in contourlet domain *n*_*i*_ is given by

$$n_{i}=\sum e_{i-k}h_{k}=e_{i}*h_{i},$$ (10)

where *e*_*i*_ is the noise component in the image domain, *h*_*i*_ is the contourlet filter coefficient, and * is the convolution operator. Since the noise in contourlet domain is a linear combination of image noise, we need to derive an equation for contourlet noise variance $\sigma_{n_{i}}^{2}$.

The noise variance of pixel *i*, denoted as $\sigma_{e_{i}}^{2}$, is equal to $E[e_{i}^{2}]$, since *E*[*e*_*i*_] = 0. Now, we derive the noise variance in contourlet domain, $\sigma_{n_{i}}^{2}$, using Eq (10) as follows:

$$\sigma_{n_{i}}^{2}=E[n_{i}^{\;2}]=E[{\left(\sum e_{i-k}h_{k}\right)}^{2}].$$ (11)

Here, the cross product terms of (∑ *e*_*i*−*k*_ *h*_*k*_)^2^ can be eliminated because neighboring noise components are assumed to be statistically independent. Thus, we obtain

$$\sigma_{n_{i}}^{2}=E[\sum (e_{i-k}{}^{2}h_{k}{}^{2})]\;=\sum \left(E[e_{i-k}{}^{2}\right]h_{k}{}^{2})=\sum \left(\sigma_{e_{i-k}}^{2}h_{k}^{2}\right)=\sigma_{e_{i}}^{2}*h_{k}^{2}.$$ (12)

As shown in Eq (12), the noise variance in contourlet domain is obtained by filtering the estimated noise variances in the image domain using the square of the contourlet filter coefficients. Finally, the denoised image can be obtained through Eq (9) using the HMM parameters and the Poisson-Gaussian noise variance in contourlet domain.

To improve noise removal performance, the Wiener filtering and cycle-spinning methods are cascaded. The Wiener filter is designed to minimize the mean squared error. Cycle spinning for noise removal is a simple and efficient method which can be applied to a shift variant transform [44,45]. The equation for cycle spinning is as follows:

$$\hat{y}=\frac{1}{K_{1}K_{2}}\;\sum_{i=1,j=1}^{K_{1},K_{2}}S_{-i,-j}(T^{-1}(D\;[T(S_{i,j}(z)])),$$ (13)

where *S*_*i*,*j*_ represents a 2-D circulant shift with *i* and *j* shifts in horizontal and vertical directions respectively. *K*_1_ and *K*_2_ are the total number of horizontal and vertical shifts, respectively. *T* represents a shift variant transform which is the contourlet transform in our case and *D* represents the presented contourlet transform based HMM denoising algorithm. The variable $\hat{y}$ is the noise removed image obtained by the cycle spinning method. Noise removed images can be obtained repeating 2-D circular shift by *K*_1_ *K*_2_ times. Consequently, the cycle spinning method improves the peak signal to noise ratio (PSNR) by averaging the noise reduced images.

**Fig 4:**
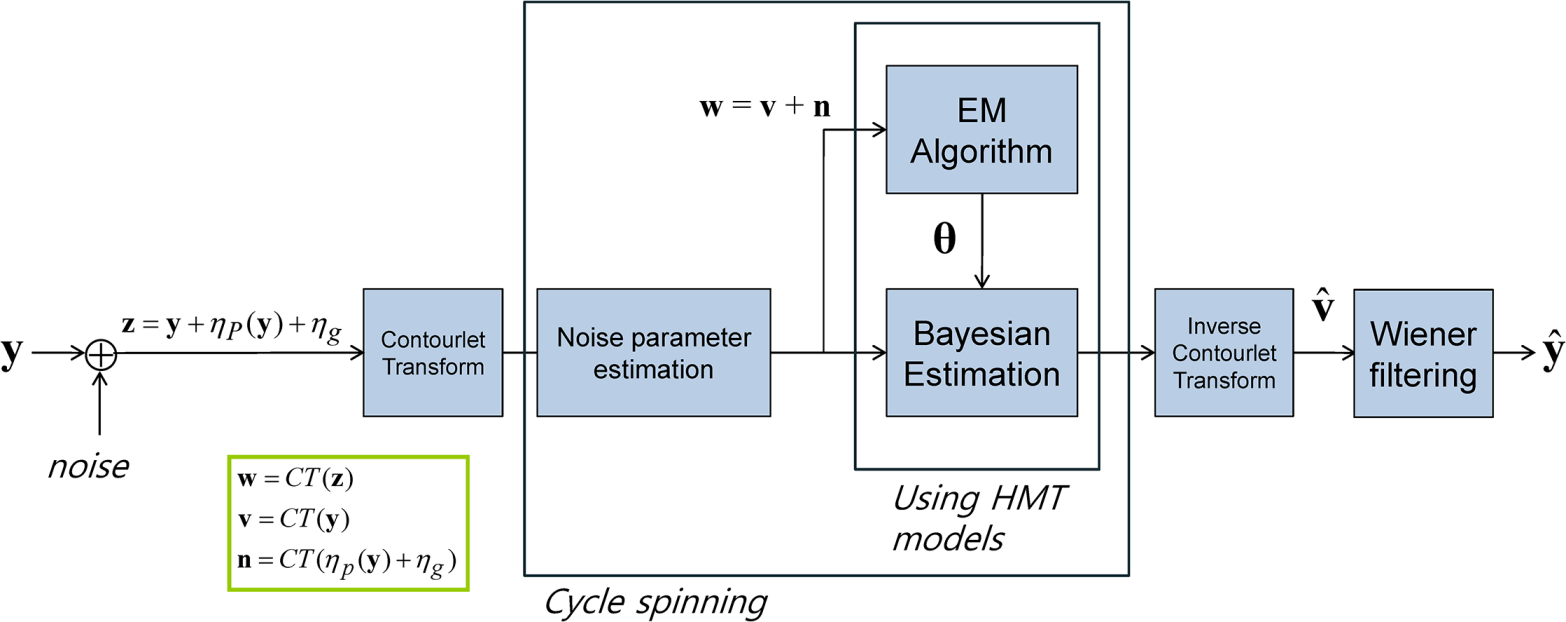
Block diagram for the proposed method (CT: contourlet transform).

A sketch of the GP-contourlet HMM denoising algorithm is illustrated in Fig 4 and presented in Algorithm 1. While the conventional HMM algorithm does not deal with signal-dependent noise, our proposed algorithm reduces Poisson-Gaussian noise successfully. Experimental results in the following section show that the proposed method shows the best performance for many images corrupted by strong Poisson noise both visually and in terms of PSNR values.

**Algorihm 1**. GP-contourlet HMM.

1. Compute GP noise parameters *a* and *b* using Eq (6).
2. Compute the variance in the image domain using Eq (4).
3. Apply the contourlet transform.

*Cycle spinning*

4 **for** *i* = 1 to *K*_1_, *j* = 1 to *K*_2_ **do**
5 Compute the variance in the transform domain using Eq (11) and the parameters, *a* and *b*, obtained at step 1.

*Expectation Maximization*

6 Estimate HMM parameter vector $\theta =\left(p_{S_{1}}(m),\varepsilon_{i,\rho (i)}^{mn},\mu_{i,m},\sigma_{i,m}^{2}\right)$ using EM algorithm.

*Bayesian estimation*

7 Estimate denoised contourlet coefficients using Eq (9).
8 **end for**
9 Apply the inverse contourlet transform to recover the denoised image.

Wiener filtering

10 Apply Wiener filtering to the denoised signal for further noise reduction.
11 Obtain the final denoised signal.

### Denoising experiments

#### Implementation of Denoising Algorithm

The Laplacian pyramid (LP) filter and the Directional filter bank (DFB) filter of the contourlet transform used in the experiments are as follows: we adopted ‘Daubeiches8’ for the LP and ‘dmaxflat5’ for the DFB [46]. We confirmed experimentally that the numbers of scales and directions in contourlet domain do not influence the performance significantly. We therefore divided the images into five scales and each scale into four directions. Grayscale images *Camera man*, *Lena*, *Boat*, *Barbara* and *Peppers* were used as test images with different noise levels. The images are available in S1 File. We first confirmed the validity of the derived Poisson noise variance in contourlet domain, and then evaluated the performance of the PG contourlet HMM noise reduction algorithm using synthetic and real data.

#### Quantitative Evaluation Measure

As a measure of quality, we used the peak signal-to-noise ratio (PSNR), defined as $PSNR=10\;{\text{log}}_{10}\left(I_{\text{max}}^{2}/MSE\right)$, where *I*_max_ is the maximum intensity of the original image and *MSE* is the mean squared error. The output MSEs of the denoised images were averaged over 10 realizations of pseudo-random noise. The standard deviation of noise was estimated from the noisy image using the noise estimation algorithm proposed by Foi *et al*. [1].

## Results and Discussion

We evaluate the performance of the PG-HMM noise reduction algorithm and apply the proposed method to confocal microscopy images in this section.

### The verification of estimated noise variance

**Fig 5:**
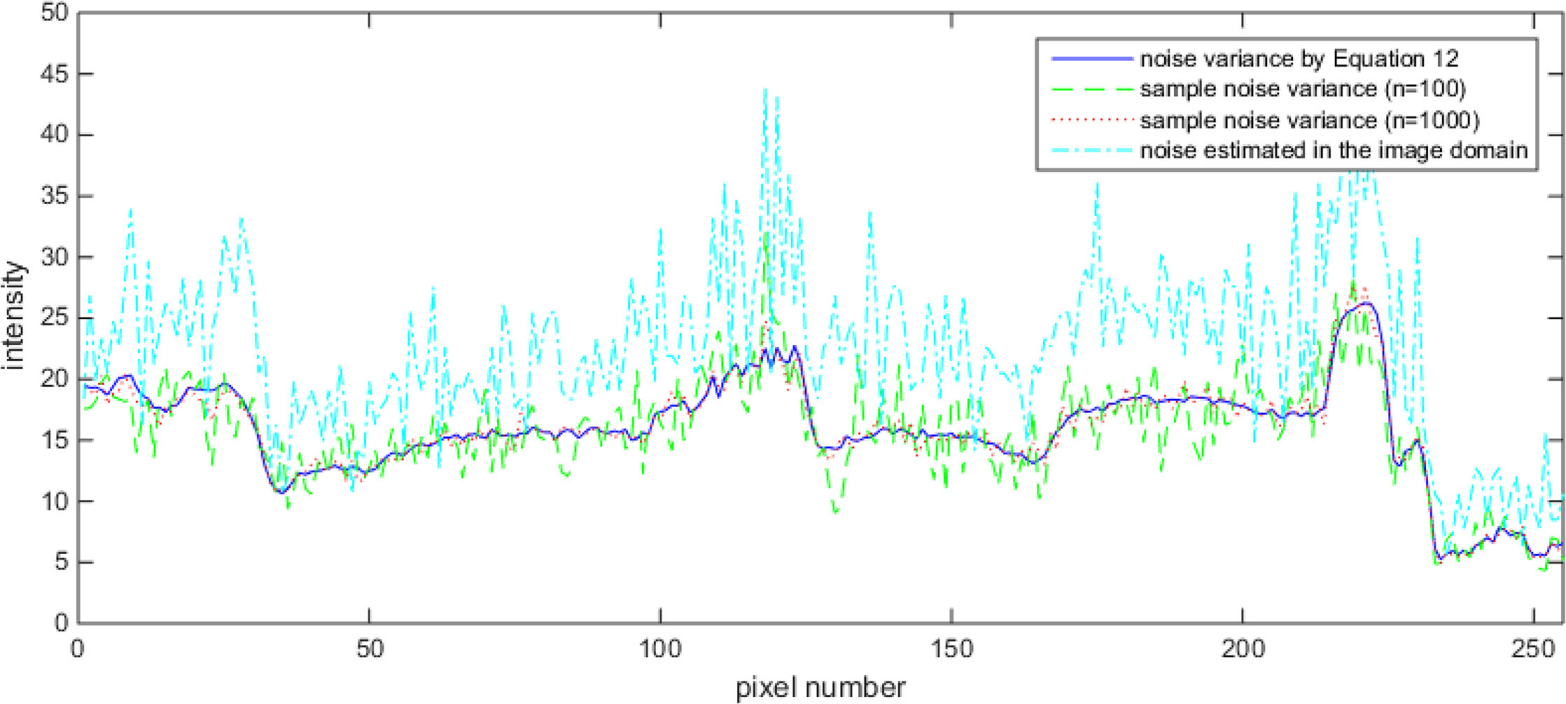
Plots of noise variances in the wavelet domain for the 50th row of the Lena image; noise variances by Eq (12) (blue solid line), sample noise variances from 100 times of noise generation (green dashed line), 1,000 times of noise generation (red dotted line) and noise variance estimated in the image domain (cyan dash-dot line).

To verify the accuracy of estimated noise variance, we performed a computer simulation using the 50th row of the *Lena* image as an example. We compare the noise variance calculated from multiple realizations of noise, which will be used as a ground truth, the noise variance estimated in the image domain and the theoretical noise variance predicted by Eq (12). The noise variance estimated in the image domain was obtained by Eq (4). In practice, the accuracy of the sample noise variance depends on the number of noise generations. The plots of simulated noise variance illustrated in Fig 5 are results of 100 times and 1000 times of noise generations. The blue solid line, green dashed line, red dotted line and cyan dash-dot line indicate the noise variance by Eq (12), the sample noise variance by Eq (11) from 100 times of noise generation, the sample noise variance by Eq (11) from 1,000 times of noise generation, and the noise variance estimated in the image domain, respectively. As shown in the plot, the result is more accurate as the number of the noise generation increases. In addition, Fig 5 demonstrates that the variance predicted by Eq (12) closely matches the noise variance estimated from multiple noise generation while it is significantly different from the image domain noise variance from Eq (4). The validity of the proposed Poisson noise estimation can be confirmed regardless of test images.

### Comparisons with existing denoising algorithms

In this section, we compare our proposed method with various denoising algorithms to show the effective denoising of our method on Poisson-Gaussian noise. In Table 1, we evaluate the performance of each block shown in Fig 4. Here, the total noise variance is fixed and only the ratio of Poisson and Gaussian noise is altered. The PSNRs of HMM based on the Gaussian noise are degraded as the Poisson component is increased. As shown in Table 1, the contourlet transform is more effective than the wavelet transform, and the cycle spinning improves the performance.

**Table 1:** Denoising performance evaluation of each block shown in Fig 4 in terms of PSNR (dB) for various noise ratio of Poisson and Gaussian components with fixed noise variance.

Image	Noise variance					
	a	0	2.65	5	5.59	5.78
	b	737.32	20^2^	10^2^	5^2^	0
Lena	Noisy image	19.46	19.46	19.47	19.47	19.45
	Gaussian wavelet HMM	29.10	28.95	28.25	27.94	27.79
	Gaussian contourlet HMM	29.45	29.28	28.51	28.22	27.91
	Gaussian contourlet HMM with modified Anscombe	29.09	28. 68	28.65	29.13	29.10
	Mixed Poisson-Gaussian contourlet Bayesian estimator	28.38	28.17	28.04	28.21	28.12
	Proposed mixed Poisson-Gaussian contourlet HMM without cycle spinning	29.71	29.54	29.47	29.32	29.46
	Proposed mixed Poisson-Gaussian contourlet HMM with cycle spinning	30.14	30.09	30.04	29.96	30.02

**Fig 6:**
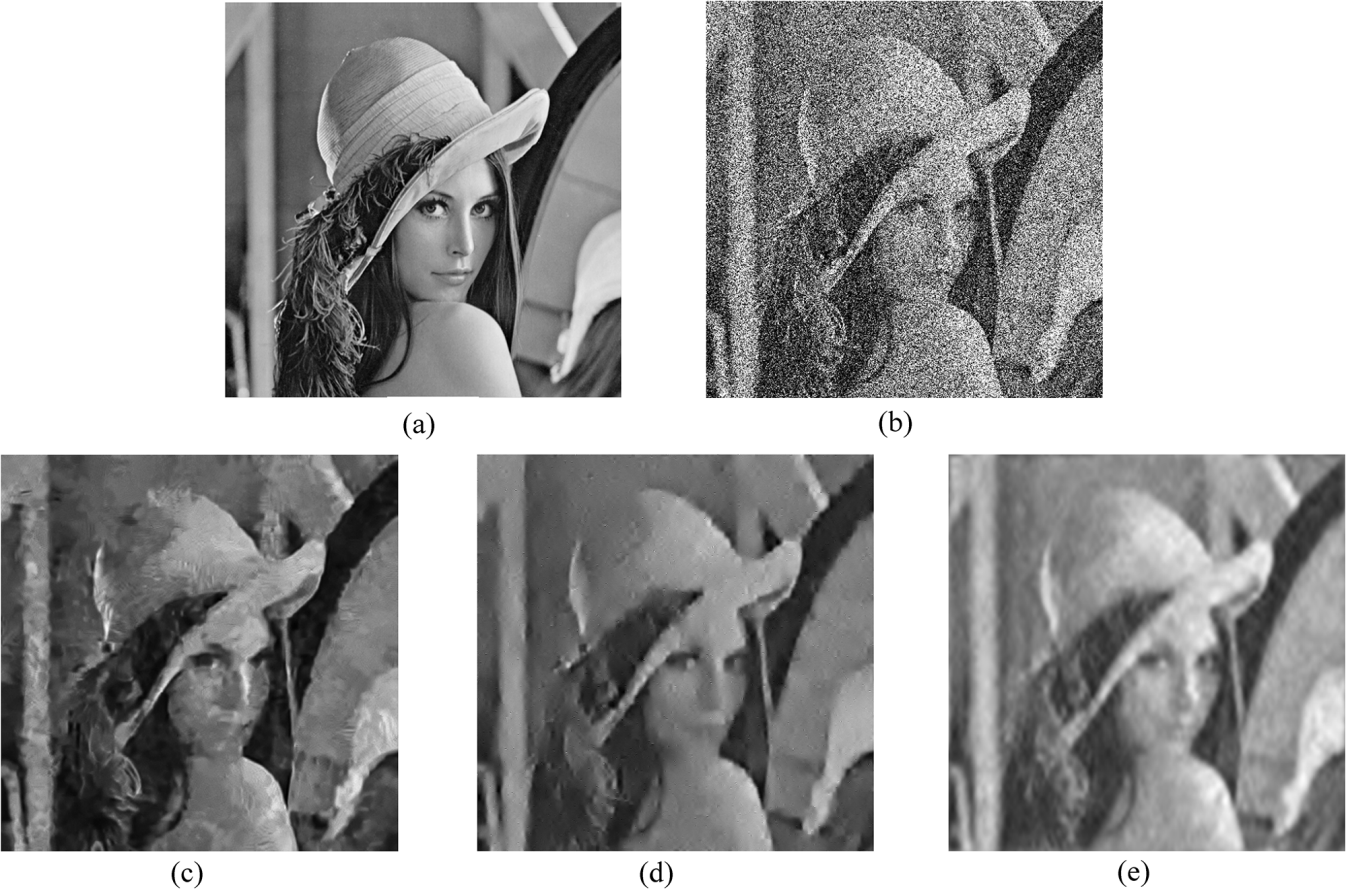
Denoising results on Lena image (http://en.wikipedia.org/wiki/File:Lenna.png.) with *a* = 1/3 and *b* = 0.3^2^. (a) original image, (b) noisy image (7.48 dB), (c) BM3D (24.80 dB), (d) PURE-LET (24.68 dB), (e) the proposed method (24.72 dB).

**Fig 7:**
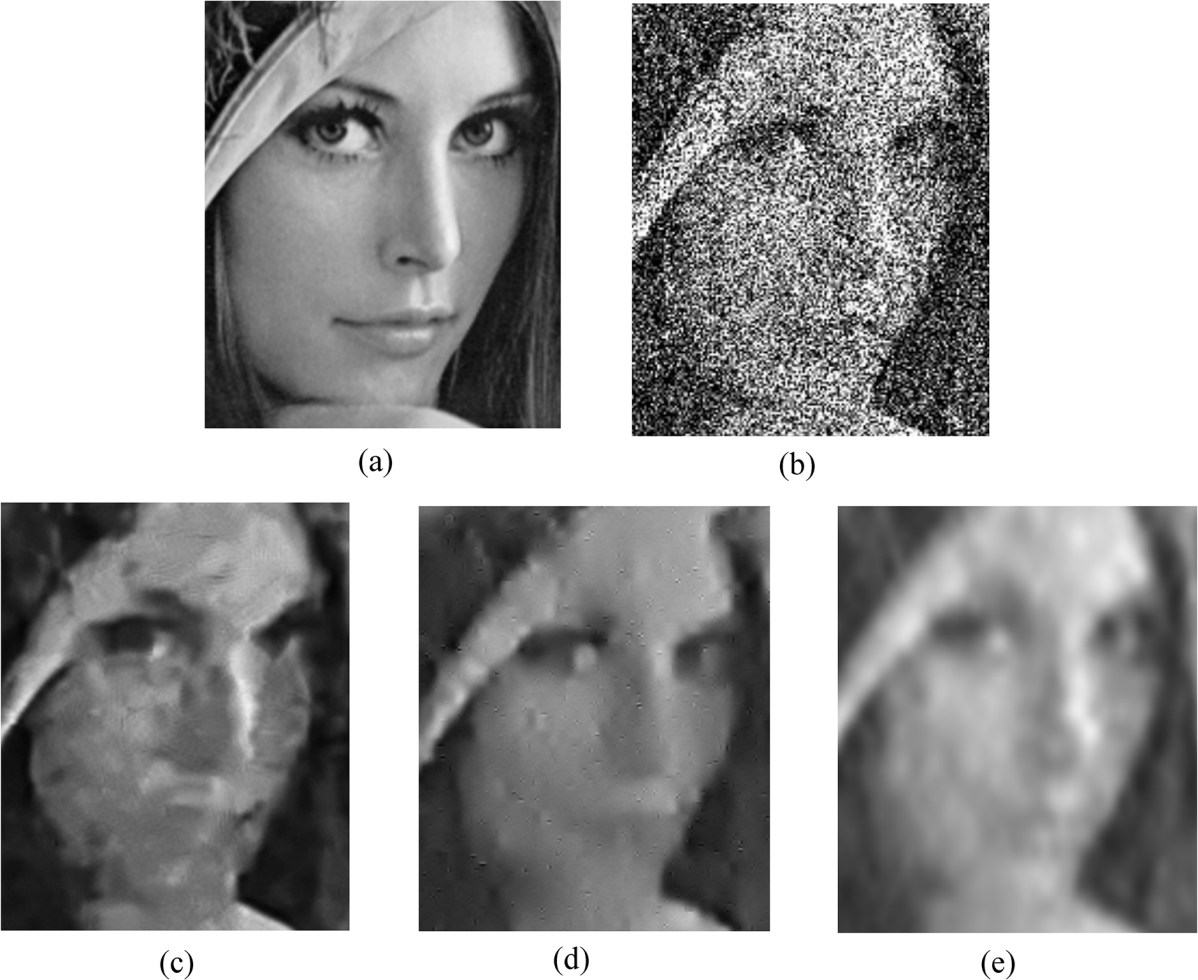
Denoising results on the magnified Lena image with *a* = 1/3 and *b* = 0.3^2^. (a) original image, (b) noisy image, (c) BM3D, (d) PURE-LET, (e) the proposed method.

Next, we compare our approach with the PURE-LET algorithm, which is a state-of-the-art denoising algorithm for Poisson-Gaussian noise, and BM3D, which is one of the most effective methods in Gaussian-based denoising algorithms. We provide comparative results of our method with the BM3D algorithm combined with the generalized Anscombe transform. The experimental results for low photon counts are shown in Table 2. Our method shows the best noise reduction for *Cameraman* and *Peppers* in low count images, while BM3D is better for *Boat* and *Lena* by a small margin. Fluorescence microscopic images are the results of photon-limited imaging, thus the performance of the algorithm for low-count images is important. In this respect, the experimental results are quite encouraging. For subjective comparison of image quality, we present the denoising results of the test images degraded by simulated Poisson noise with peak intensity 3 and Gaussian noise with standard deviation 0.3 in Figs 6 and 7. Although all three methods have similar PSNRs, Fig 7 shows that the proposed method provides fewer visual artifacts with a smoother face and preserves edges better than the PURE-LET or BM3D techniques. Our method restores the nose and mouth of the face for a more natural look and does not demolish the boundary as shown in Fig 6. To give the reader an indication of the typical computation times, for Cameraman (512x512) the various denoising methods require time approximately as follows: BM3D with GAT 2.5 s, UWT/BDCT PURE-LET 13.5 s, and the proposed method 10.2 s. The compared algorithms were implemented in MATLAB and BM3D is a pre-compiled execution file. These results are obtained with an Intel Core i7-3770 processor, running at 3.4 GHz.

**Table 2:** Comparison of the proposed method with the BM3D method and PURE-LET in terms of PSNR (dB) for various low-count cases.

Image	Noise variance					
	a	1	1/2	1/3	1/5	1/10
	b	0.1^2^	0.2^2^	0.3^2^	0.5^2^	1^2 0^
Camera man	Noisy image	3.26	6.20	8.84	10.85	13.34
	BM3D with GAT	19.76	21.44	22.43	23.79	**25.52**
	UWT/BDCT PURE-LET	**20.44**	21.61	22.27	23.36	24.74
	Poisson-Gaussian contourlet HMM	19.59	**22.59**	**23.06**	**24.43**	25.16
Peppers	Noisy image	2.72	5.66	7.28	9.36	12.01
	BM3D with GAT	21.94	23.54	24.55	25.82	27.39
	UWT/BDCT PURE-LET	**22.31**	**23.68**	24.35	25.55	26.91
	Poisson-Gaussian contourlet HMM	19.16	23.05	**24.77**	**26.00**	**27.55**
Barbara	Noisy image	3.29	6.19	7.85	9.90	12.47
	BM3D with GAT	20.72	**22.22**	23.03	**24.17**	**25.83**
	UWT/BDCT PURE-LET	**20.93**	21.60	22.08	22.56	23.48
	Poisson-Gaussian contourlet HMM	20.14	21.93	**23.05**	23.82	24.80
Boat	Noisy image	2.33	5.29	6.94	9.03	11.69
	BM3D with GAT	20.99	**22.65**	**23.15**	**24.20**	25.65
	UWT/BDCT PURE-LET	**21.38**	22.38	22.92	23.80	25.17
	Poisson-Gaussian contourlet HMM	20.22	22.45	23.00	24.12	**25.72**
Lena	Noisy image	2.89	5.82	7.48	9.52	12.17
	BM3D with GAT	22.72	**24.37**	**24.80**	**26.14**	**27.64**
	UWT/BDCT PURE-LET	**23.23**	24.30	24.68	25.80	27.23
	Poisson-Gaussian contourlet HMM	22.19	23.17	24.72	25.88	26.98

### Application to fluorescence microscopy images

**Fig 8:**
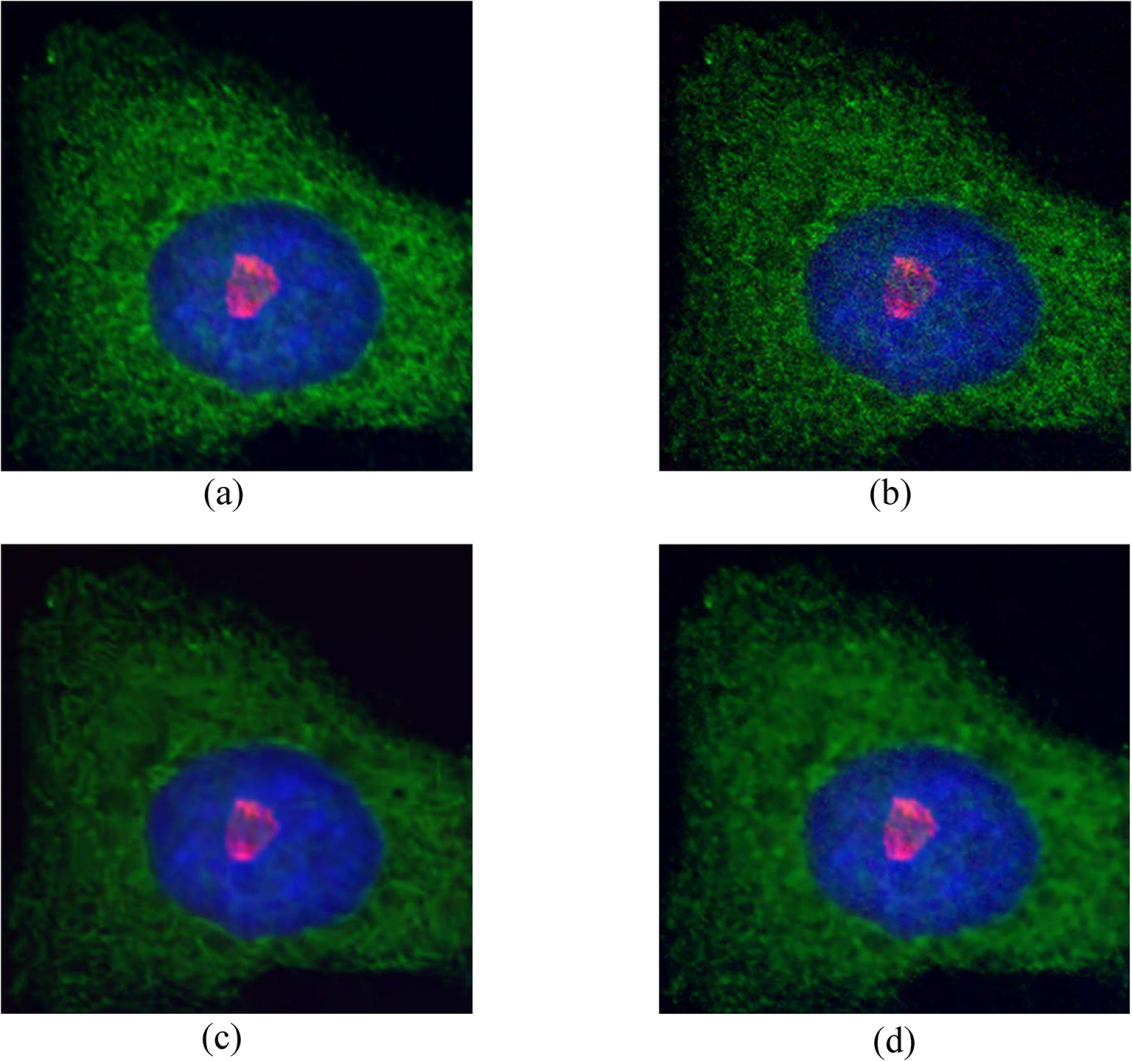
Denoising results on the first set of HeLa cell images. (a) an average of 100 images used as the ground truth, (b) single acquisition image, (c) BM3D, (d) the proposed method.

In this section, we present denoising results of photon-limited fluorescence microscopy images. Our proposed method and the BM3D method are applied to two fluorescence microscopy image sets. The first set of images was acquired from a Nikon C1 Plus confocal laser microscope at the Medicinal Bioconvergence Research Center at Seoul National University. The data set contained 100 images with a 512x512 size of fixed HeLa cells, labeled with three fluorescent dyes: Alexafluor555 in the red channel, Alexafluor488 in the green channel, and DAPI in the blue channel. The average of 100 images is used as the baseline for PSNR calculation. The data set is available in S2 File. The visual quality of the cell image is evaluated from Fig 8. Fig 8A and 8B presents the baseline and the observed data with single acquisition, respectively. Fig 8C and 8D shows the denoised images from the BM3D and the proposed method, respectively. As observed in Fig 8, the proposed algorithm significantly reduces the level of noise and still recovered the details in the green channel. The comparison of PSNR between the proposed method and the BM3D method in each channel is presented in Table 3. Our proposed method performs better than the BM3D method, except for the blue channel. The BM3D is preferable in the blue channel as it is particularly effective with periodic textures or flat regions.

**Table 3:** Comparison of the proposed method with the BM3D method in terms of PSNR (dB) for each channel on the first set of HeLa cell images.

	R channel	G channel	B channel	total
Noisy image	28.64 dB	21.95 dB	28.41dB	26.33 dB
BM3D with GAT	32.69 dB	25.23 dB	**39.97 dB**	32.63 dB
Poisson-Gaussian contourlet HMM	**35.58 dB**	**29.53 dB**	36.94 dB	**34.02 dB**

**Fig 9:**
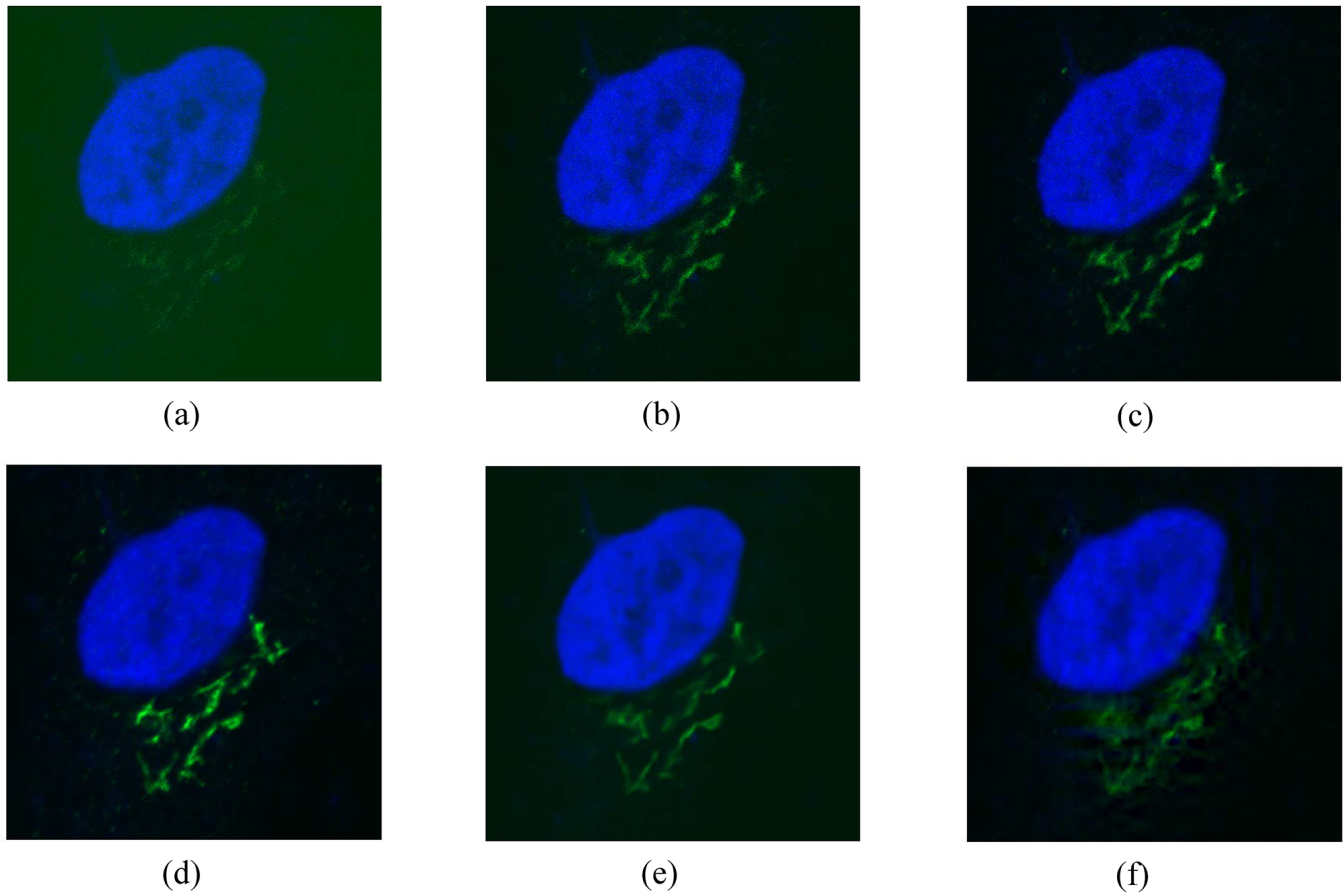
The second set of HeLa cell images and denoising results. First low: the second set of HeLa cell images with different laser intensities. (a) 0.2, (b) 0.4, (c) 0.8. Second low: denoising results on HeLa cell image with laser intensity 0.4. (d) an average of 40 images used as the ground truth, (e) BM3D, (f) the proposed method.

The second data set was obtained from a Nikon A1R confocal laser microscope at the Department of Life Science of Ewha W. University. The data set contains 40 images of fixed HeLa cells of 512x512 size, labeled with two fluorescent dyes: golgin97 in the green channel, and DAPI in the blue channel. An average of the 40 images is used as the baseline, which is presented in Fig 9(D). The data set is available in S3 File. To evaluate the denoising performance for different noise levels, we obtained three image sets with different laser intensities as shown in Fig 9A–9C. The denoising results for the single acquisition image with laser intensity 0.4 are presented in Fig 9D–9F. The PSNR results are provided in Table 4. While the proposed algorithm is more effective than the BM3D when the laser intensity is weak, the BM3D is more effective than our proposed method when the laser intensity increases.

**Table 4:** Comparison of the proposed method with the BM3D method in terms of PSNR(dB) for various laser intensities on the second set of HeLa cell images.

	Laser intensity		
	0.2	0.4	0.8
Estimated *a* when *b* = 0	27.33	9.35	3.95
BM3D with modified Anscombe	14.86 dB	23.21 dB	**29.11 dB**
Proposed contourlet HMM with Bayesian estimation	**16.12 dB**	**23.44 dB**	27.64 dB

## Conclusions

In this paper, an effective denoising algorithm for mixed Poisson-Gaussian noise in low-count images is presented. We applied the contourlet transform for sparse representation of signal, and adopted the HMM for effective signal modeling in the transform domain. The contourlet transform not only separates of images into high and low frequency components but also provides information about the directional components in the images using the Laplacian pyramid filter and the directional filter bank. The HMM algorithm adopts an independent mixture model to match the non-Gaussian nature of the contourlet coefficients and adopts hidden Markov models to characterize the key dependencies between the contourlet coefficients. Furthermore, this method estimates optimal HMM parameters using the EM algorithm. The Poisson-Gaussian noise variance in contourlet domain is obtained by filtering the noise variance of each pixel with the square of the contourlet filter coefficients. Using the estimated HMM parameters of the signal and noise variances, the signal-dependent noise is reduced through Bayesian estimation.

We finally show the experimental results with simulations and fluorescence microscopy images and demonstrate the improved performance of the proposed approach when photon count is limited through extensive comparisons with the traditional Bayesian estimator and state-of-the-art techniques. We demonstrate that the performance of the proposed method, which is based on accurate source pdf modeling with HMM and Gaussian mixture, is comparable to those of high performance denoising methods such as BM3D combined with the modified Anscombe transform or PURE-LET. Our approach has the following advantages. First, noise variance in the transform domain is estimated more accurately using the convolution. Second, the noise reduction performance is superior in very low-count images. Third, the proposed method show good performance for the images with irregular patterns such as cell images. Although the computation time is not low compared with existing methods, it can be shortened by modifying HMM training process. The denoising performance of the proposed method can be improved further by incorporating the nonlocal means algorithm, since our method is based on a point-wise technique. The proposed method can be modified for feature detection or segmentation as well as for denoising 1-D signals.

## Supporting Information

S1 Fig *Lena* image. (TIF)

S1 File Test image data set. *Camera man* (**Figure A**), *Boat* (**Figure B**), *Barbara* (**Figure C**) and *Peppers* (**Figure D**)
(ZIP)

S2 File The first data set of HeLa cell images. (ZIP)

S3 File The second data set of HeLa cell images. (ZIP)
