# Supplementary figures and images for: Poisson-Gaussian Noise Reduction Using the Hidden Markov Model in Contourlet Domain for Fluorescence Microscopy Images (part 1 of 2)
Source: PLoS One. 2015 Sep 9;10(9):e0136964. doi: 10.1371/journal.pone.0136964 (PMC4564212; doi:10.1371/journal.pone.0136964)

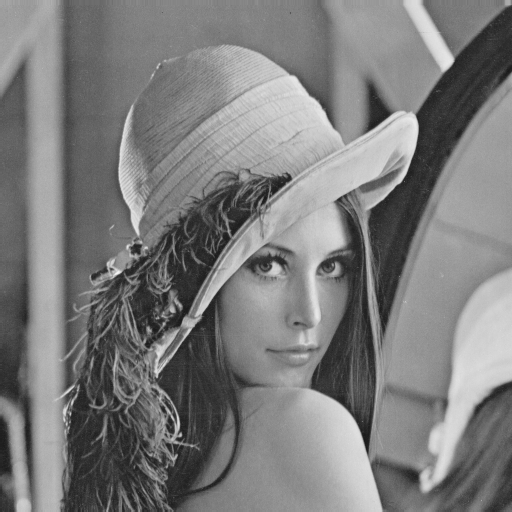

Supplement: S1 Fig — (TIF) [file pone.0136964.s001.tif]

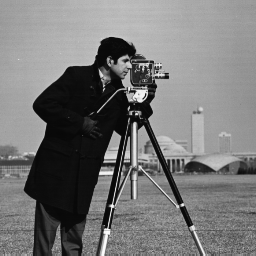

Supplement: S1 File — Camera man (Figure A), Boat (Figure B), Barbara (Figure C) and Peppers (Figure D) (ZIP) [file pone.0136964.s002.zip › S1_File/Figure_A.tif]

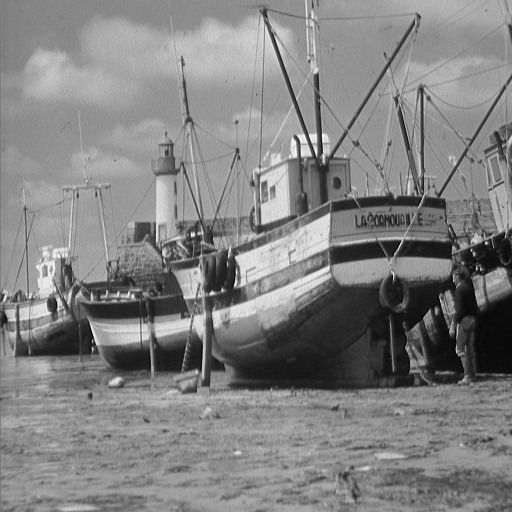

Supplement: S1 File — Camera man (Figure A), Boat (Figure B), Barbara (Figure C) and Peppers (Figure D) (ZIP) [file pone.0136964.s002.zip › S1_File/Figure_B.png]

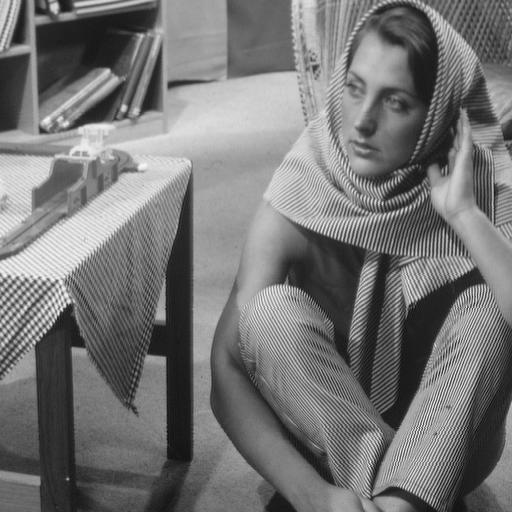

Supplement: S1 File — Camera man (Figure A), Boat (Figure B), Barbara (Figure C) and Peppers (Figure D) (ZIP) [file pone.0136964.s002.zip › S1_File/Figure_C.TIF]

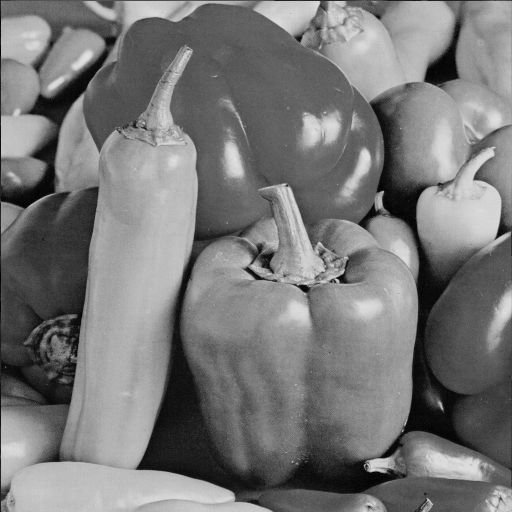

Supplement: S1 File — Camera man (Figure A), Boat (Figure B), Barbara (Figure C) and Peppers (Figure D) (ZIP) [file pone.0136964.s002.zip › S1_File/Figure_D.tif]

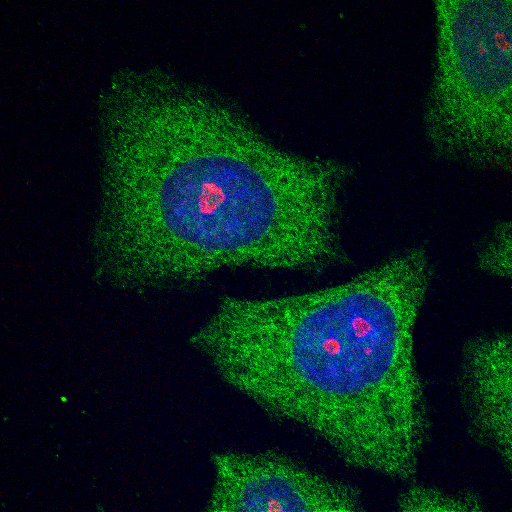

Supplement: S2 File — (ZIP) [file pone.0136964.s003.zip › S2_File/1.tif]

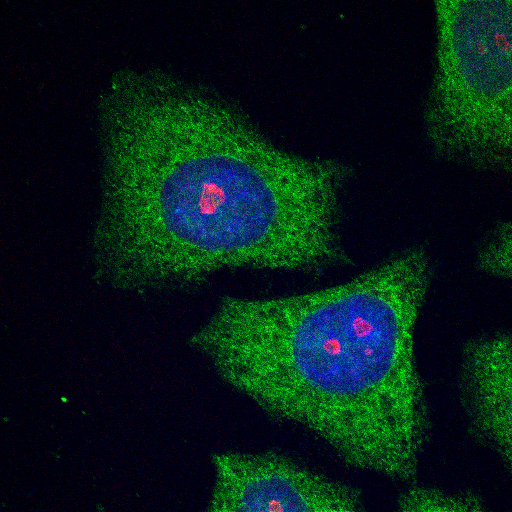

Supplement: S2 File — (ZIP) [file pone.0136964.s003.zip › S2_File/10.tif]

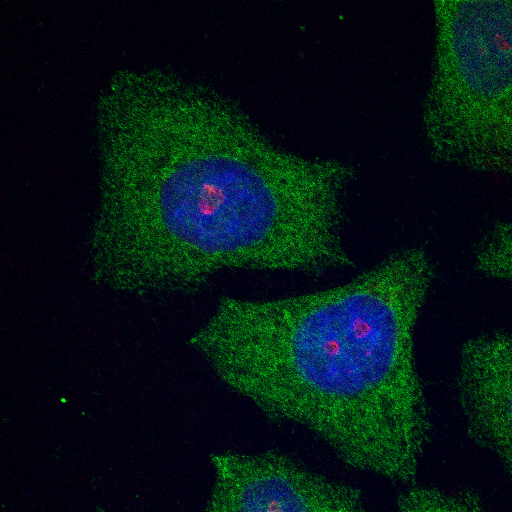

Supplement: S2 File — (ZIP) [file pone.0136964.s003.zip › S2_File/100.tif]

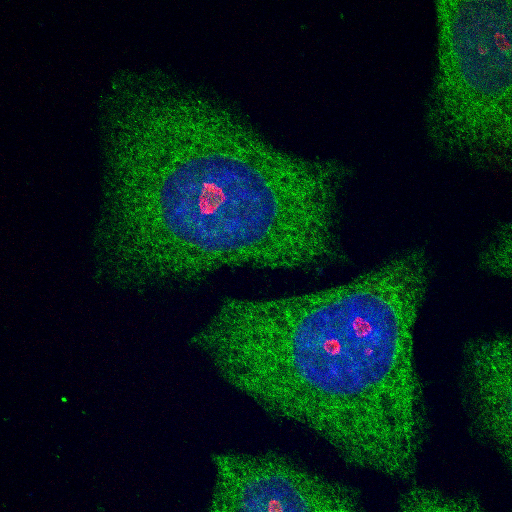

Supplement: S2 File — (ZIP) [file pone.0136964.s003.zip › S2_File/11.tif]

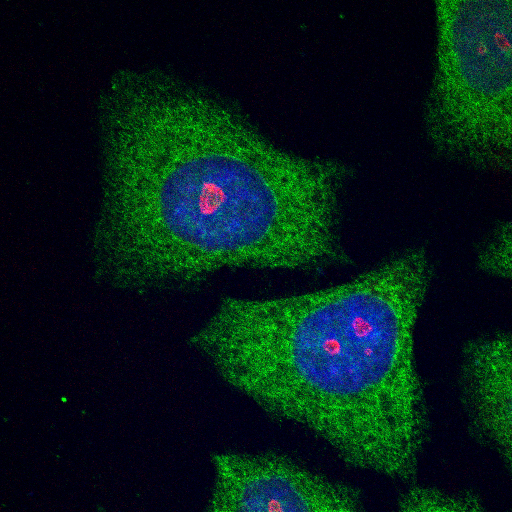

Supplement: S2 File — (ZIP) [file pone.0136964.s003.zip › S2_File/12.tif]

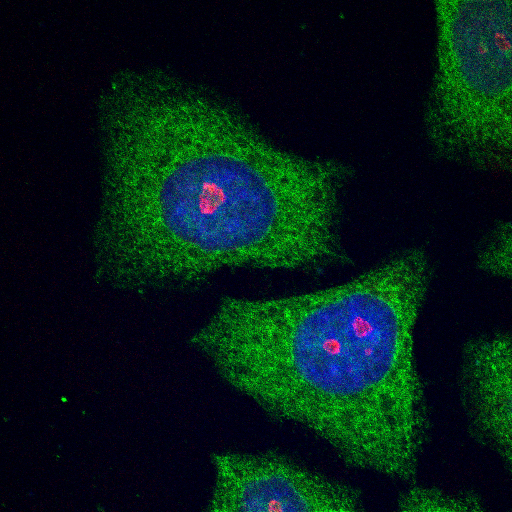

Supplement: S2 File — (ZIP) [file pone.0136964.s003.zip › S2_File/13.tif]

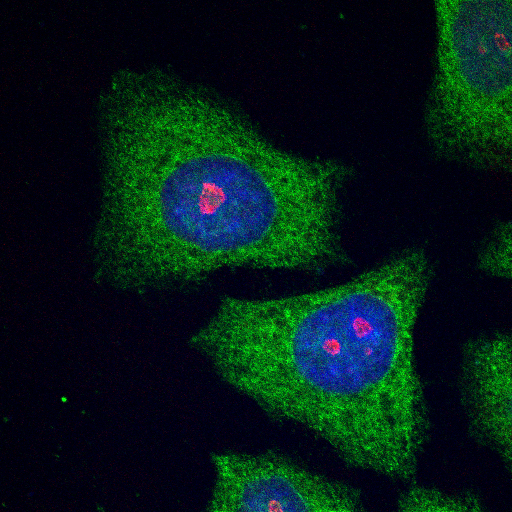

Supplement: S2 File — (ZIP) [file pone.0136964.s003.zip › S2_File/14.tif]

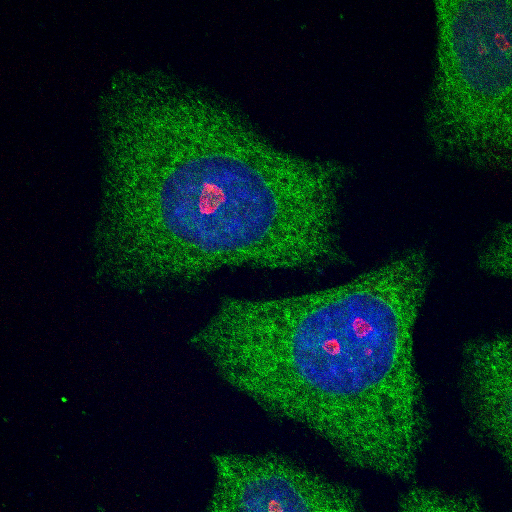

Supplement: S2 File — (ZIP) [file pone.0136964.s003.zip › S2_File/15.tif]

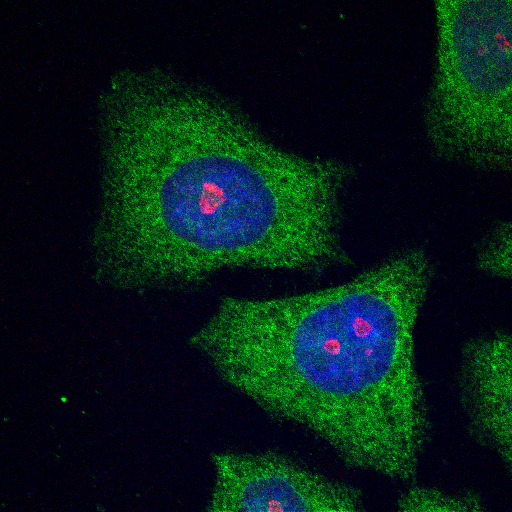

Supplement: S2 File — (ZIP) [file pone.0136964.s003.zip › S2_File/16.tif]

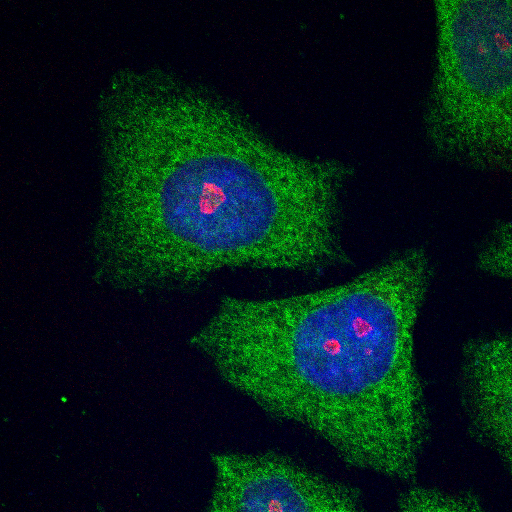

Supplement: S2 File — (ZIP) [file pone.0136964.s003.zip › S2_File/17.tif]

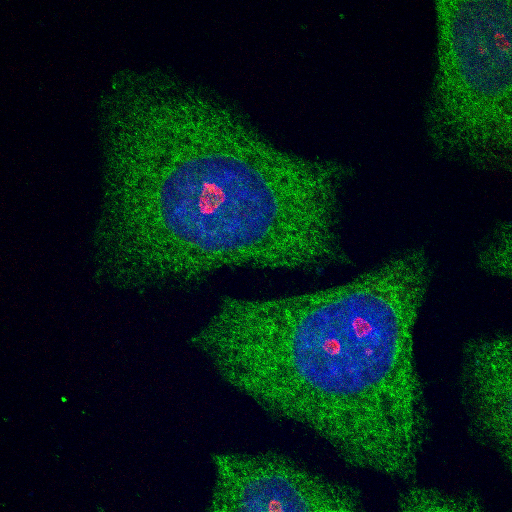

Supplement: S2 File — (ZIP) [file pone.0136964.s003.zip › S2_File/18.tif]

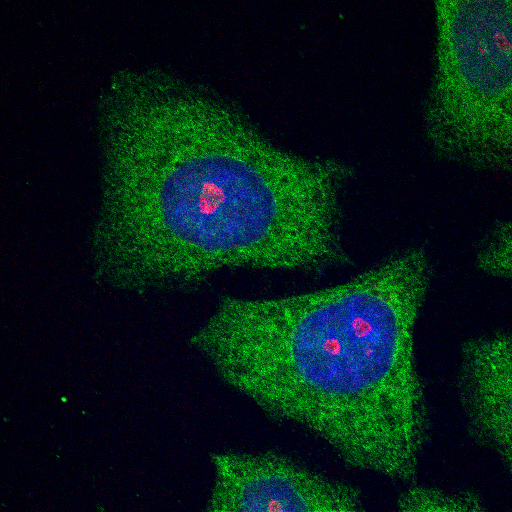

Supplement: S2 File — (ZIP) [file pone.0136964.s003.zip › S2_File/19.tif]

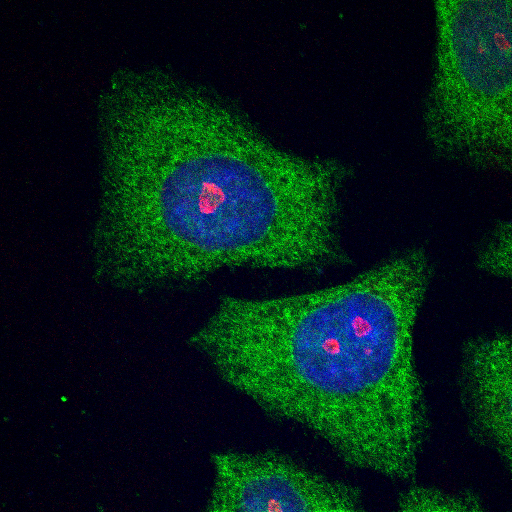

Supplement: S2 File — (ZIP) [file pone.0136964.s003.zip › S2_File/2.tif]

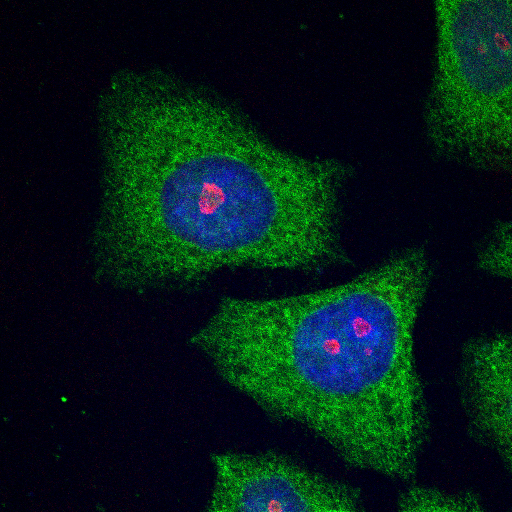

Supplement: S2 File — (ZIP) [file pone.0136964.s003.zip › S2_File/20.tif]

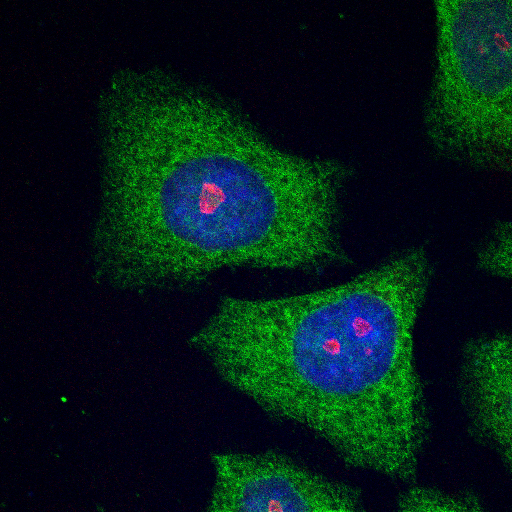

Supplement: S2 File — (ZIP) [file pone.0136964.s003.zip › S2_File/21.tif]

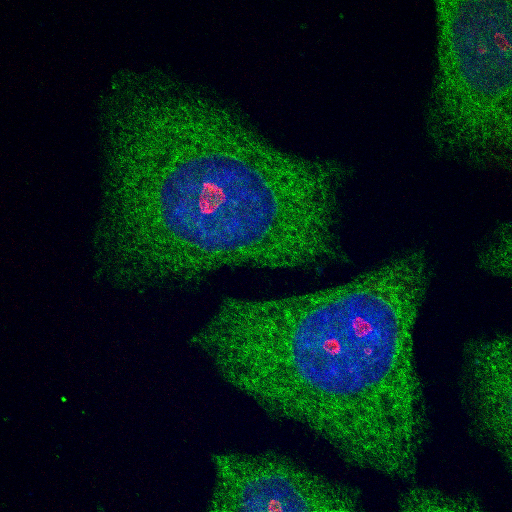

Supplement: S2 File — (ZIP) [file pone.0136964.s003.zip › S2_File/22.tif]

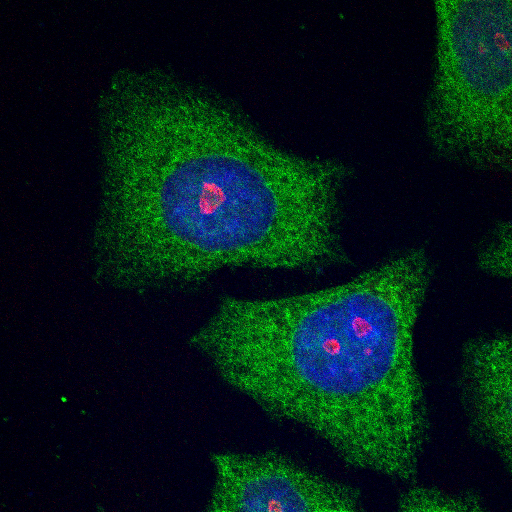

Supplement: S2 File — (ZIP) [file pone.0136964.s003.zip › S2_File/23.tif]

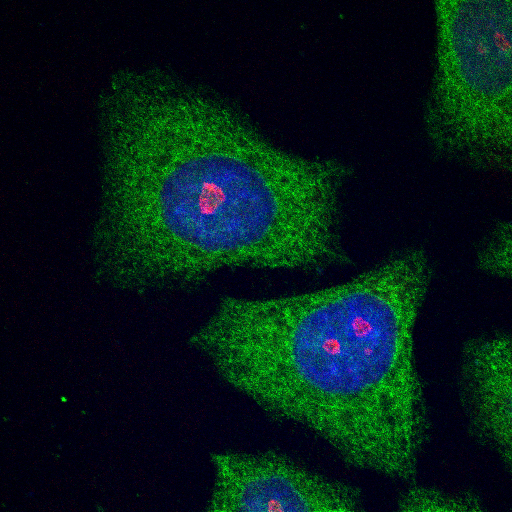

Supplement: S2 File — (ZIP) [file pone.0136964.s003.zip › S2_File/24.tif]

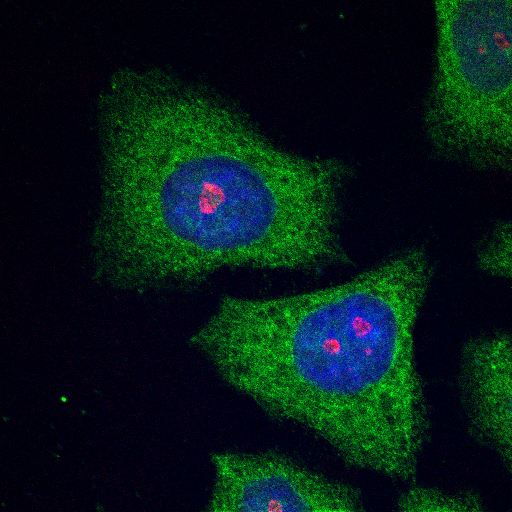

Supplement: S2 File — (ZIP) [file pone.0136964.s003.zip › S2_File/25.tif]

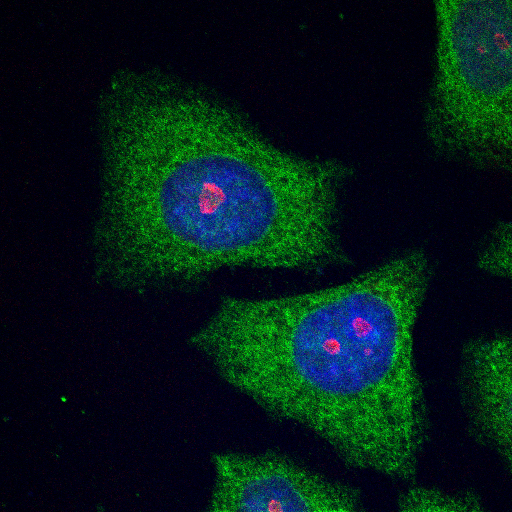

Supplement: S2 File — (ZIP) [file pone.0136964.s003.zip › S2_File/26.tif]

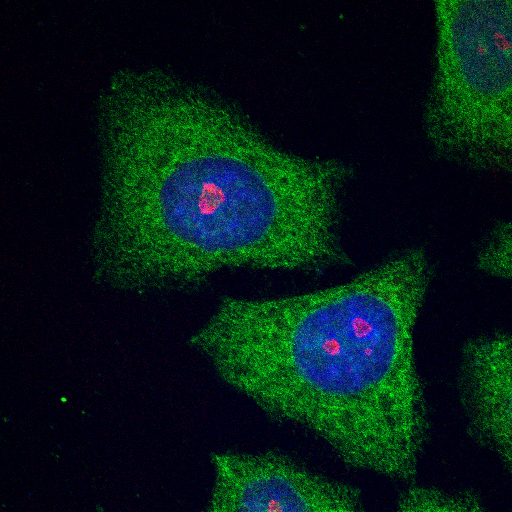

Supplement: S2 File — (ZIP) [file pone.0136964.s003.zip › S2_File/27.tif]

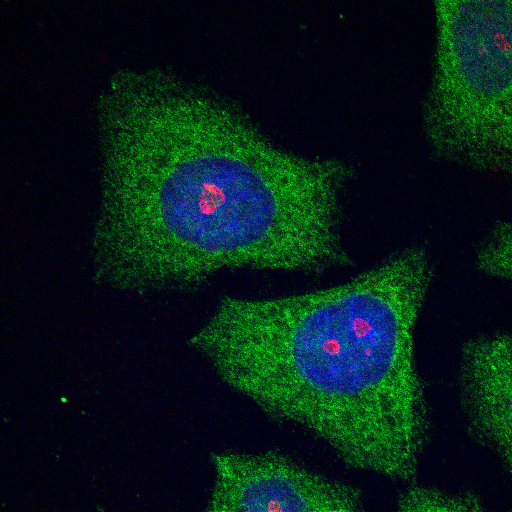

Supplement: S2 File — (ZIP) [file pone.0136964.s003.zip › S2_File/28.tif]

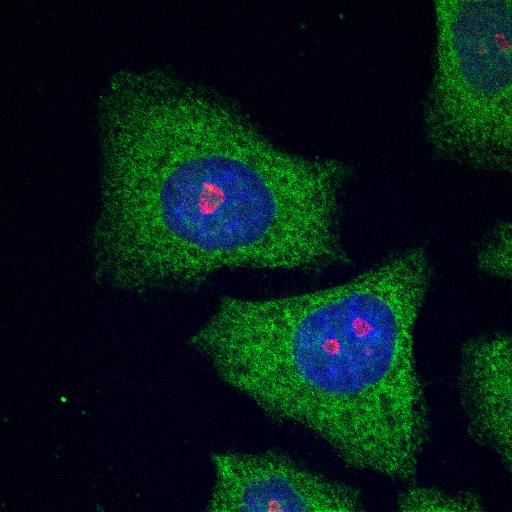

Supplement: S2 File — (ZIP) [file pone.0136964.s003.zip › S2_File/29.tif]

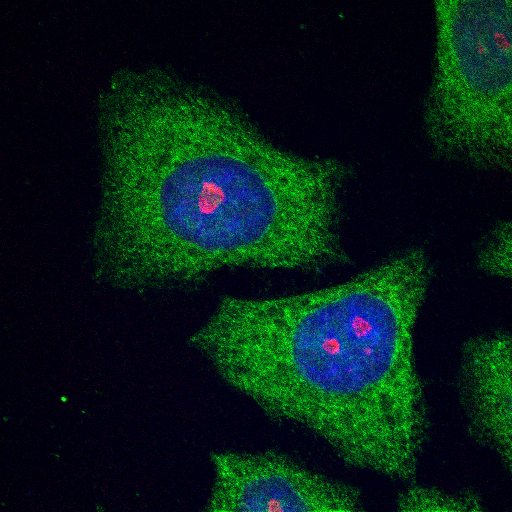

Supplement: S2 File — (ZIP) [file pone.0136964.s003.zip › S2_File/3.tif]

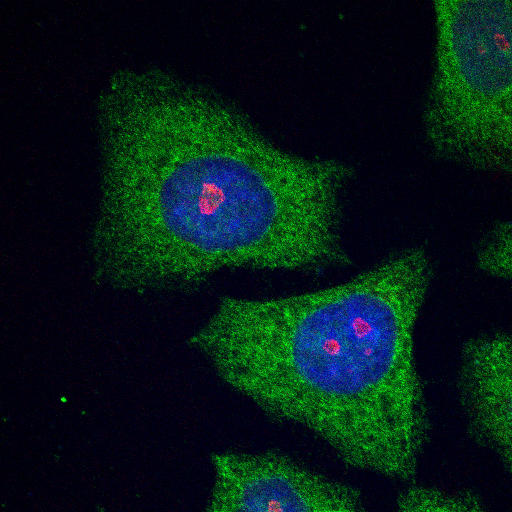

Supplement: S2 File — (ZIP) [file pone.0136964.s003.zip › S2_File/30.tif]

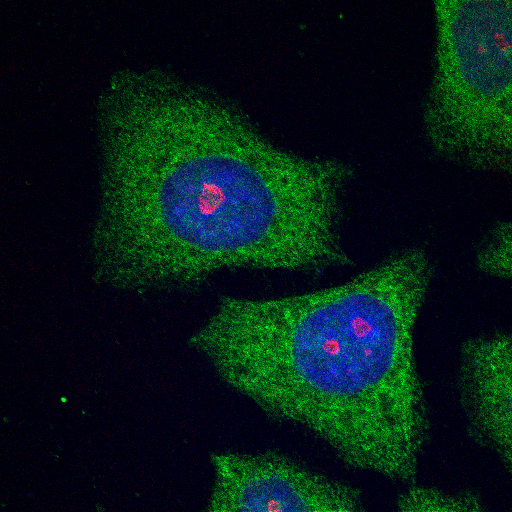

Supplement: S2 File — (ZIP) [file pone.0136964.s003.zip › S2_File/31.tif]

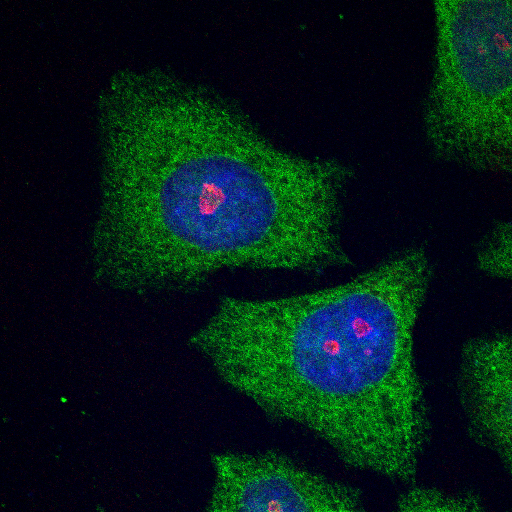

Supplement: S2 File — (ZIP) [file pone.0136964.s003.zip › S2_File/32.tif]

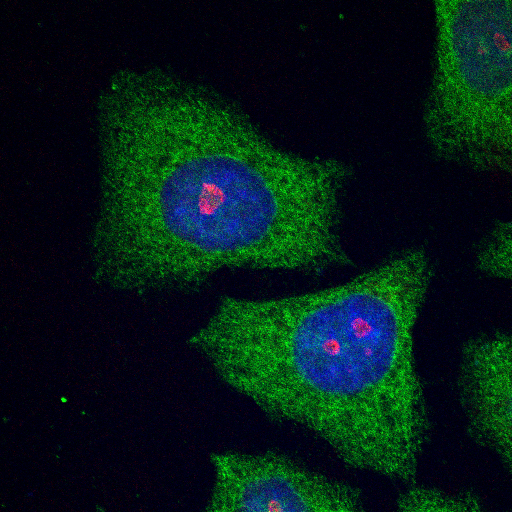

Supplement: S2 File — (ZIP) [file pone.0136964.s003.zip › S2_File/33.tif]

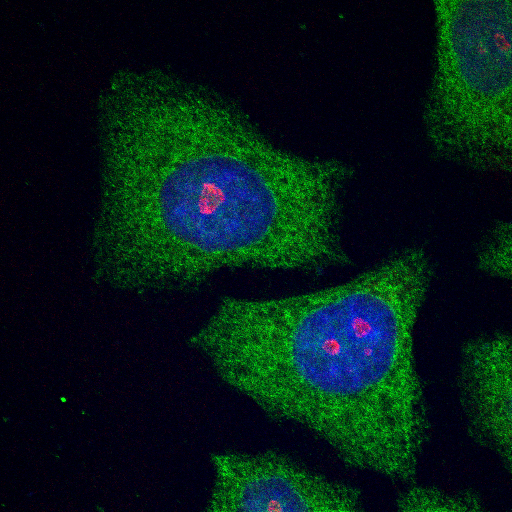

Supplement: S2 File — (ZIP) [file pone.0136964.s003.zip › S2_File/34.tif]

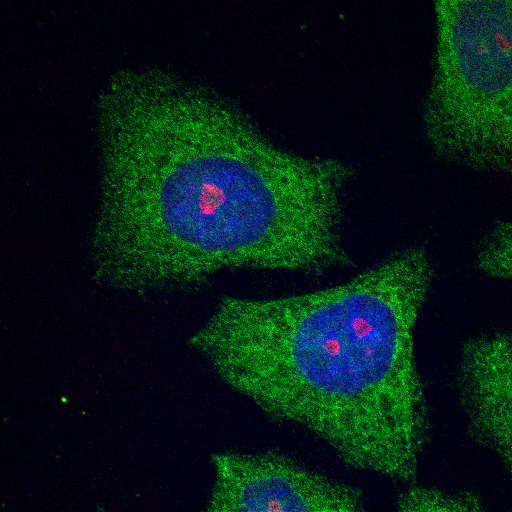

Supplement: S2 File — (ZIP) [file pone.0136964.s003.zip › S2_File/35.tif]

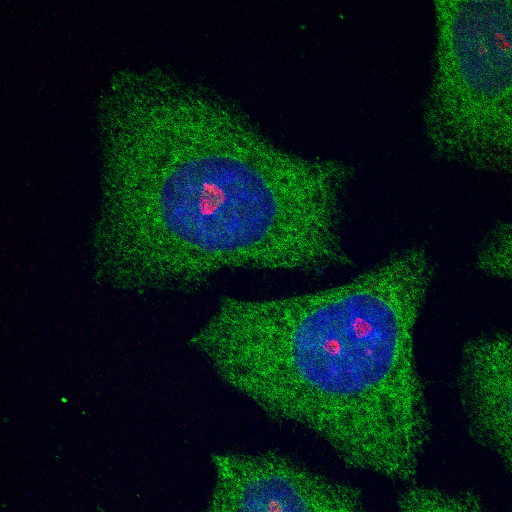

Supplement: S2 File — (ZIP) [file pone.0136964.s003.zip › S2_File/36.tif]

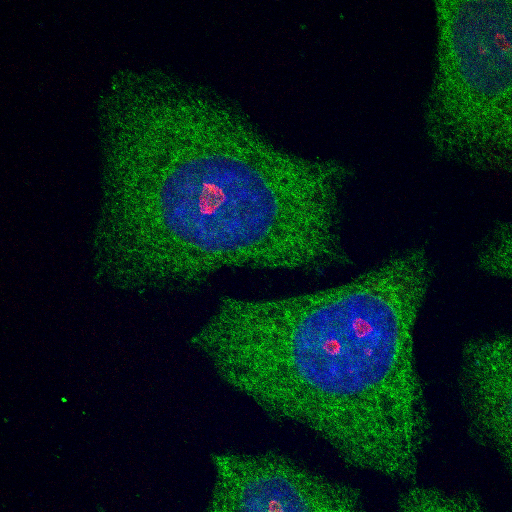

Supplement: S2 File — (ZIP) [file pone.0136964.s003.zip › S2_File/37.tif]

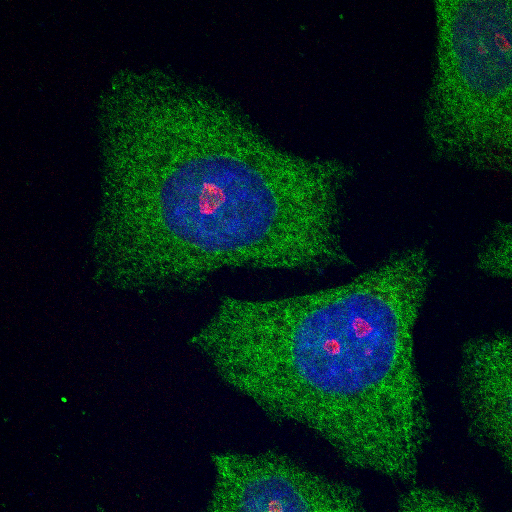

Supplement: S2 File — (ZIP) [file pone.0136964.s003.zip › S2_File/38.tif]

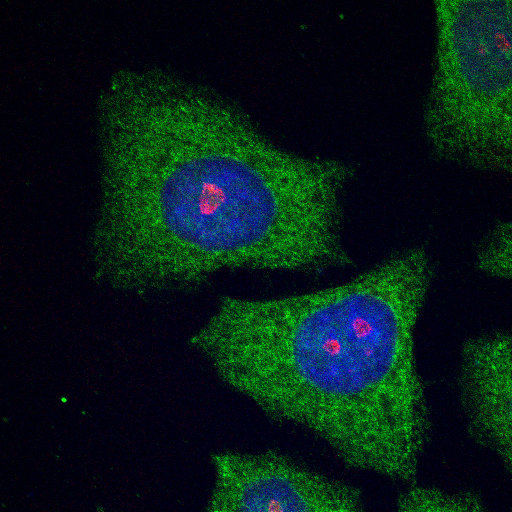

Supplement: S2 File — (ZIP) [file pone.0136964.s003.zip › S2_File/39.tif]

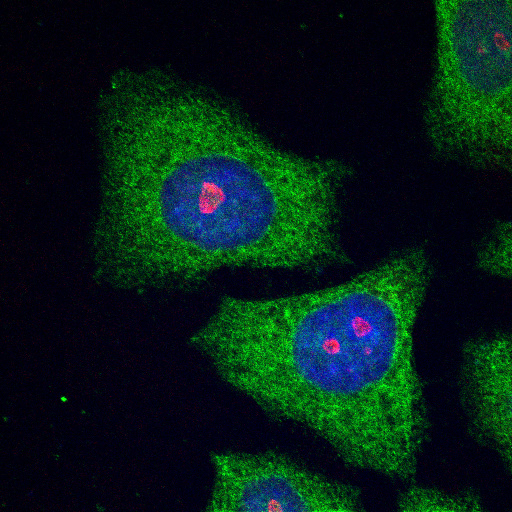

Supplement: S2 File — (ZIP) [file pone.0136964.s003.zip › S2_File/4.tif]

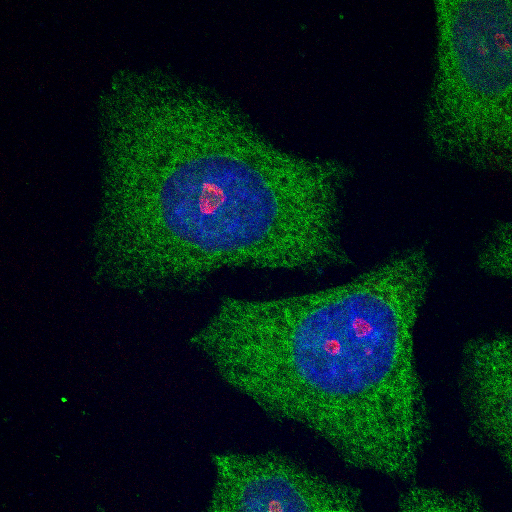

Supplement: S2 File — (ZIP) [file pone.0136964.s003.zip › S2_File/40.tif]

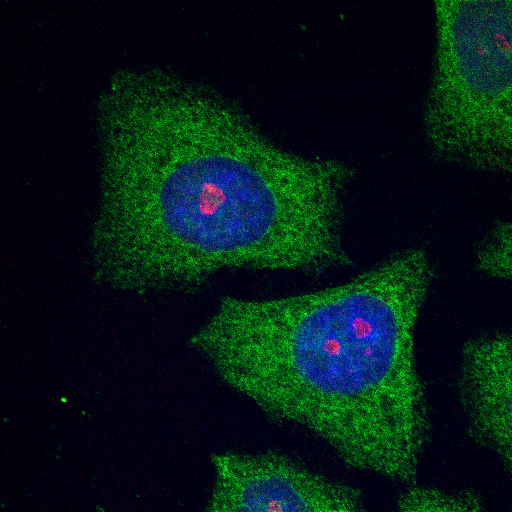

Supplement: S2 File — (ZIP) [file pone.0136964.s003.zip › S2_File/41.tif]

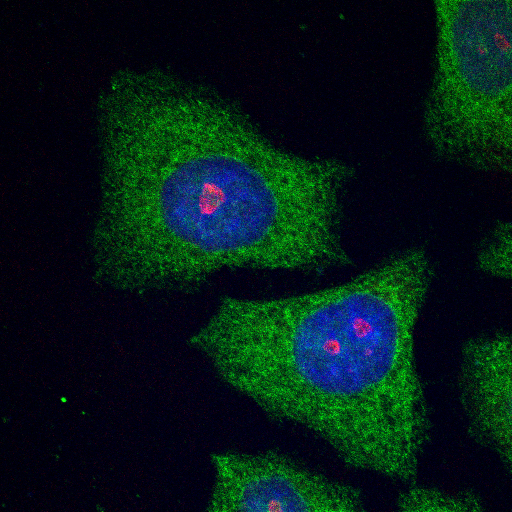

Supplement: S2 File — (ZIP) [file pone.0136964.s003.zip › S2_File/42.tif]

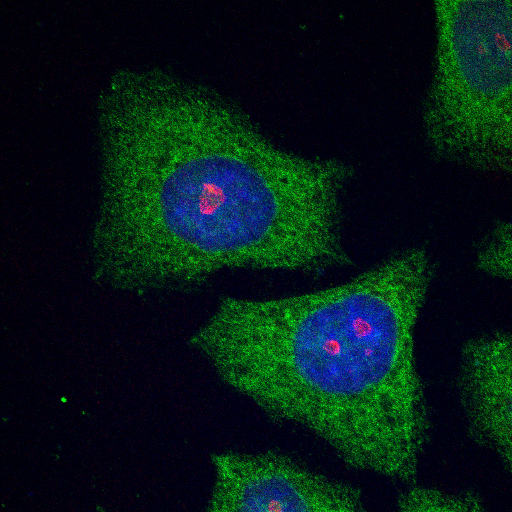

Supplement: S2 File — (ZIP) [file pone.0136964.s003.zip › S2_File/43.tif]

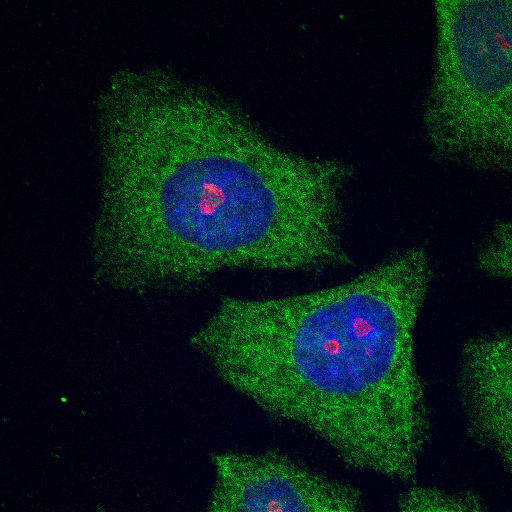

Supplement: S2 File — (ZIP) [file pone.0136964.s003.zip › S2_File/44.tif]

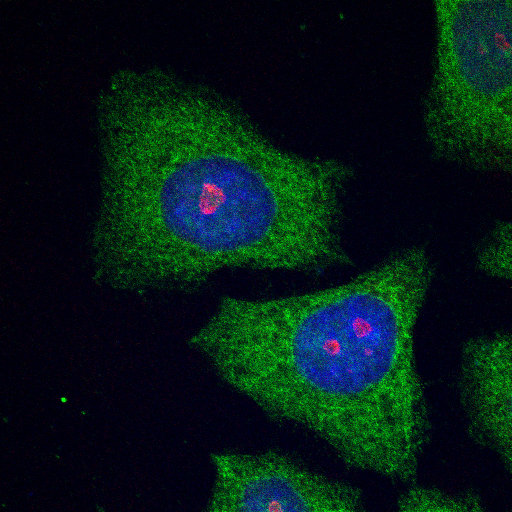

Supplement: S2 File — (ZIP) [file pone.0136964.s003.zip › S2_File/45.tif]

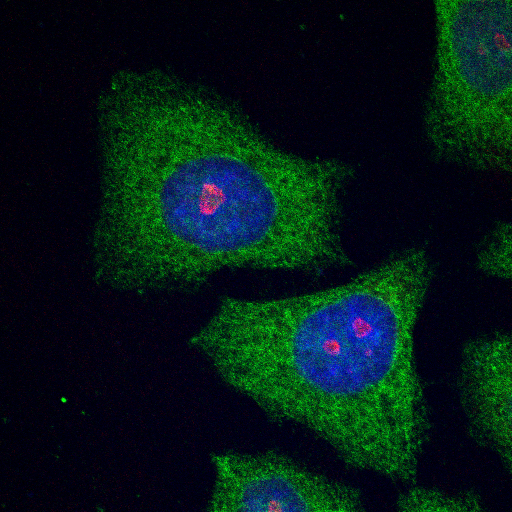

Supplement: S2 File — (ZIP) [file pone.0136964.s003.zip › S2_File/46.tif]

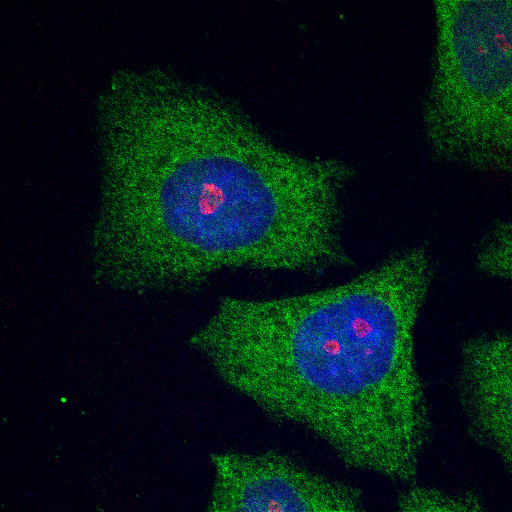

Supplement: S2 File — (ZIP) [file pone.0136964.s003.zip › S2_File/47.tif]

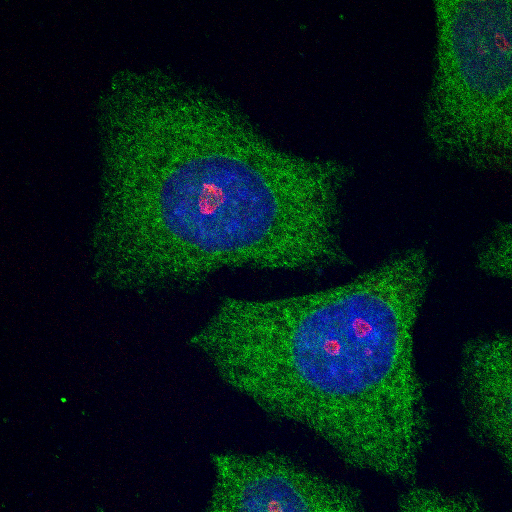

Supplement: S2 File — (ZIP) [file pone.0136964.s003.zip › S2_File/48.tif]

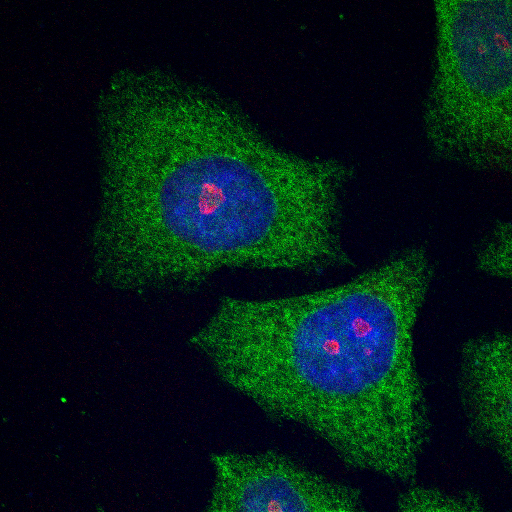

Supplement: S2 File — (ZIP) [file pone.0136964.s003.zip › S2_File/49.tif]

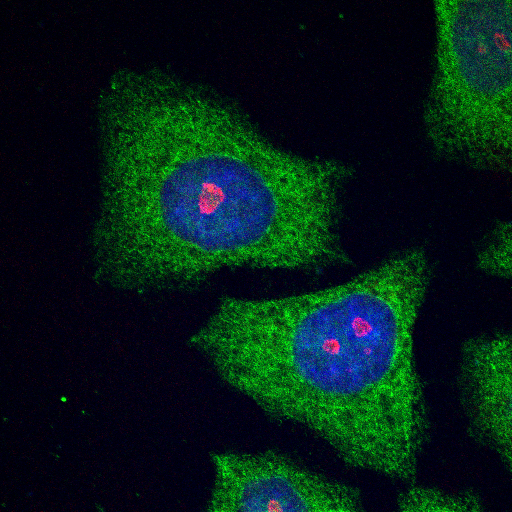

Supplement: S2 File — (ZIP) [file pone.0136964.s003.zip › S2_File/5.tif]

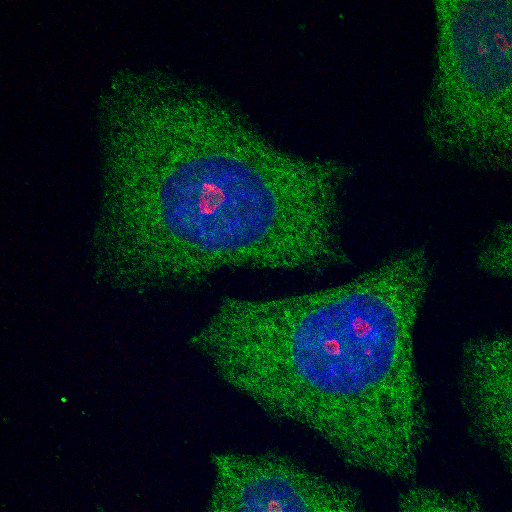

Supplement: S2 File — (ZIP) [file pone.0136964.s003.zip › S2_File/50.tif]

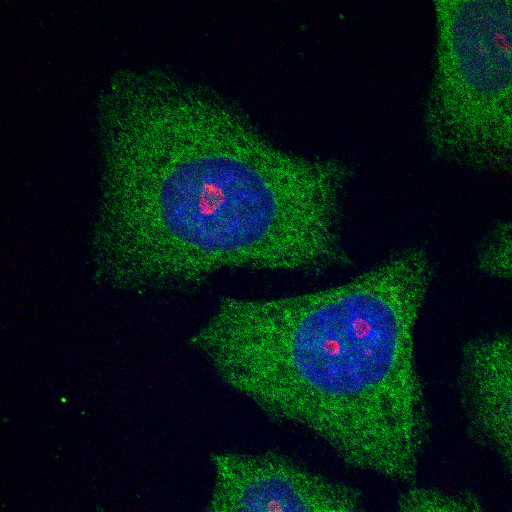

Supplement: S2 File — (ZIP) [file pone.0136964.s003.zip › S2_File/51.tif]

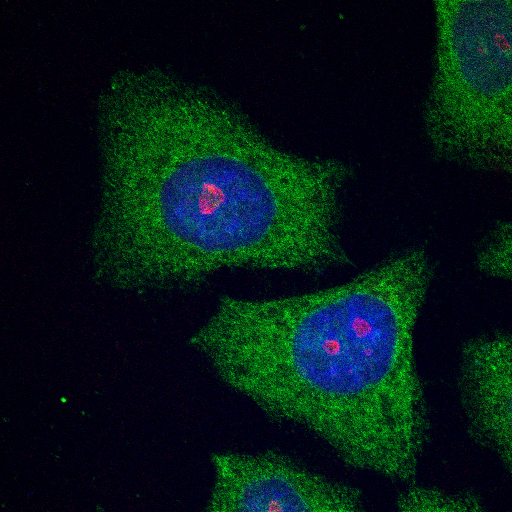

Supplement: S2 File — (ZIP) [file pone.0136964.s003.zip › S2_File/52.tif]

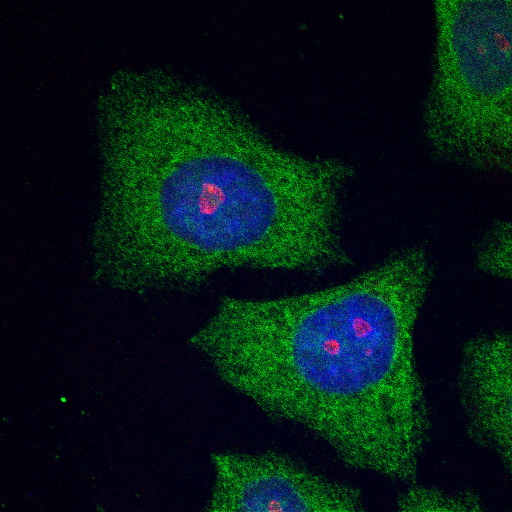

Supplement: S2 File — (ZIP) [file pone.0136964.s003.zip › S2_File/53.tif]

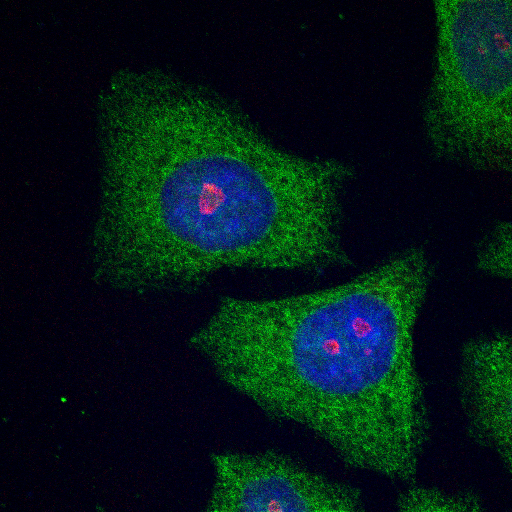

Supplement: S2 File — (ZIP) [file pone.0136964.s003.zip › S2_File/54.tif]

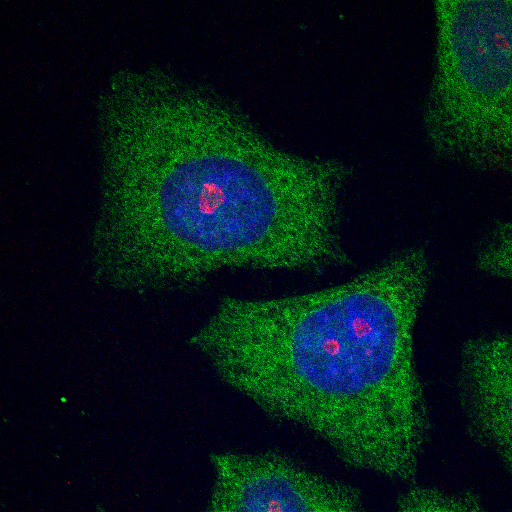

Supplement: S2 File — (ZIP) [file pone.0136964.s003.zip › S2_File/55.tif]

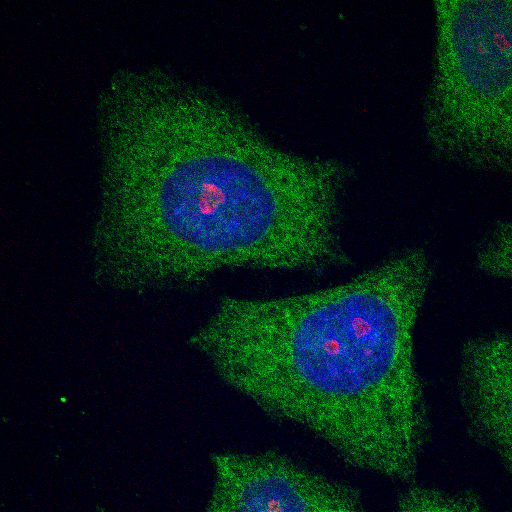

Supplement: S2 File — (ZIP) [file pone.0136964.s003.zip › S2_File/56.tif]

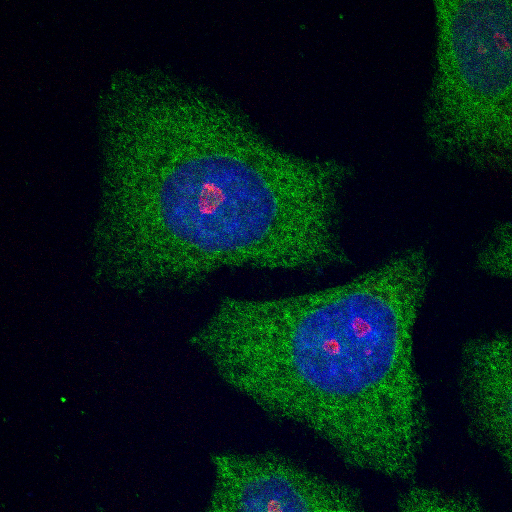

Supplement: S2 File — (ZIP) [file pone.0136964.s003.zip › S2_File/57.tif]

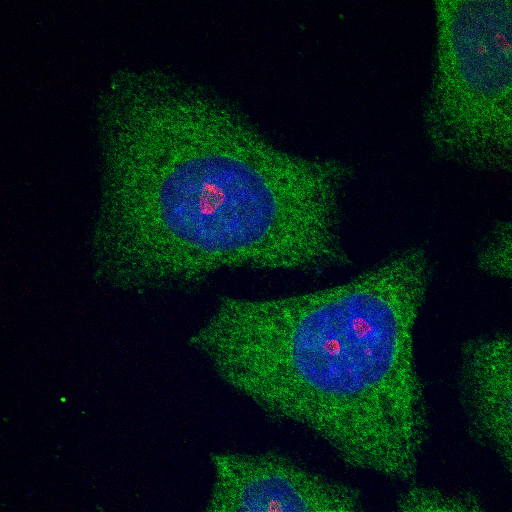

Supplement: S2 File — (ZIP) [file pone.0136964.s003.zip › S2_File/58.tif]

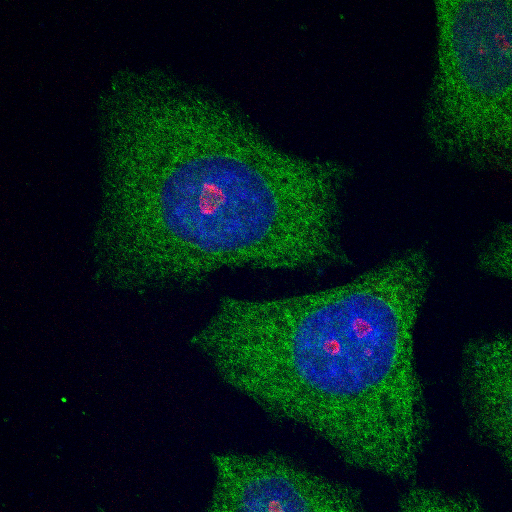

Supplement: S2 File — (ZIP) [file pone.0136964.s003.zip › S2_File/59.tif]

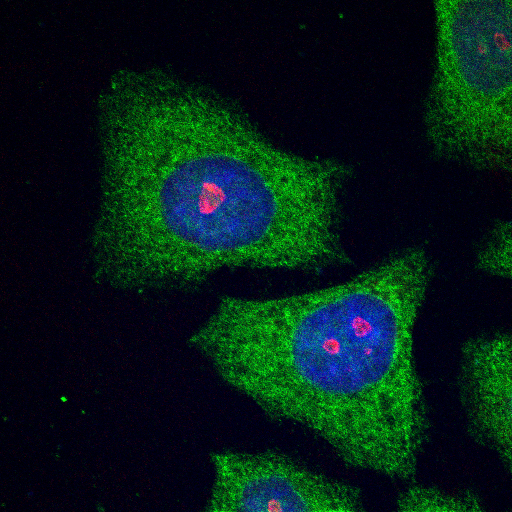

Supplement: S2 File — (ZIP) [file pone.0136964.s003.zip › S2_File/6.tif]

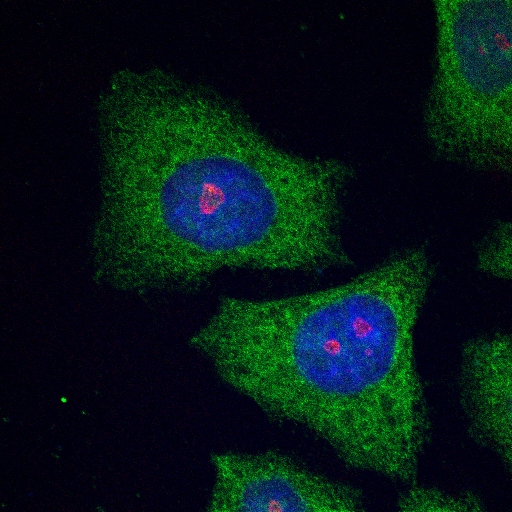

Supplement: S2 File — (ZIP) [file pone.0136964.s003.zip › S2_File/60.tif]

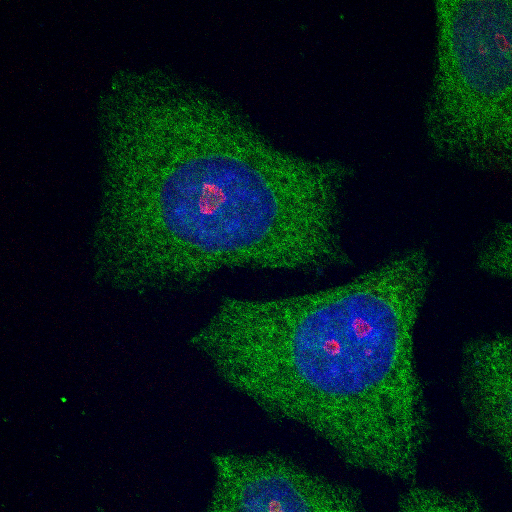

Supplement: S2 File — (ZIP) [file pone.0136964.s003.zip › S2_File/61.tif]

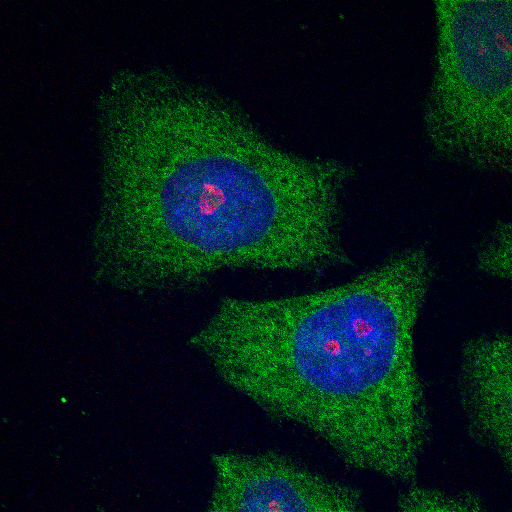

Supplement: S2 File — (ZIP) [file pone.0136964.s003.zip › S2_File/62.tif]

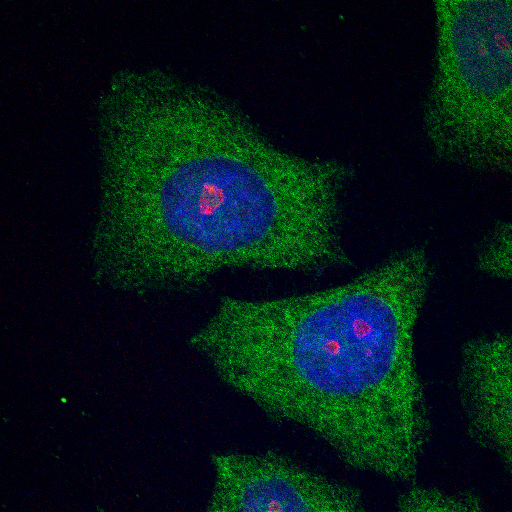

Supplement: S2 File — (ZIP) [file pone.0136964.s003.zip › S2_File/63.tif]

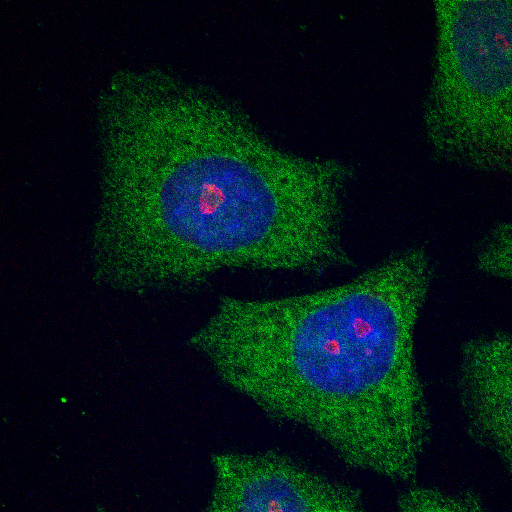

Supplement: S2 File — (ZIP) [file pone.0136964.s003.zip › S2_File/64.tif]

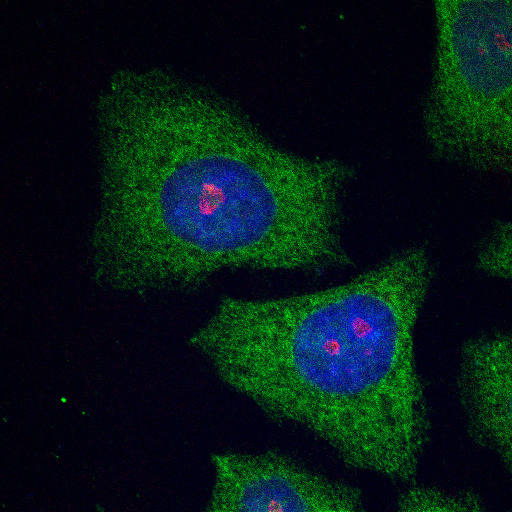

Supplement: S2 File — (ZIP) [file pone.0136964.s003.zip › S2_File/65.tif]

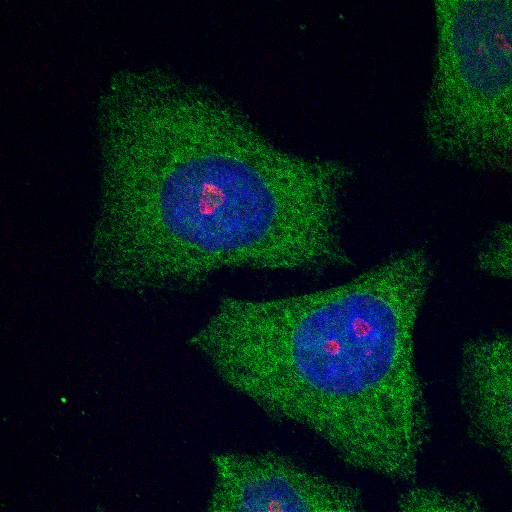

Supplement: S2 File — (ZIP) [file pone.0136964.s003.zip › S2_File/66.tif]

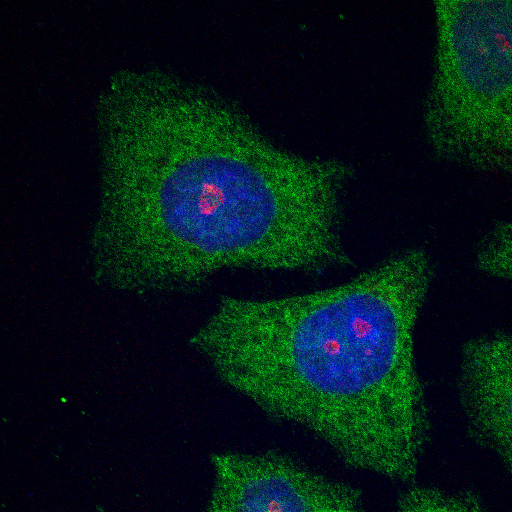

Supplement: S2 File — (ZIP) [file pone.0136964.s003.zip › S2_File/67.tif]

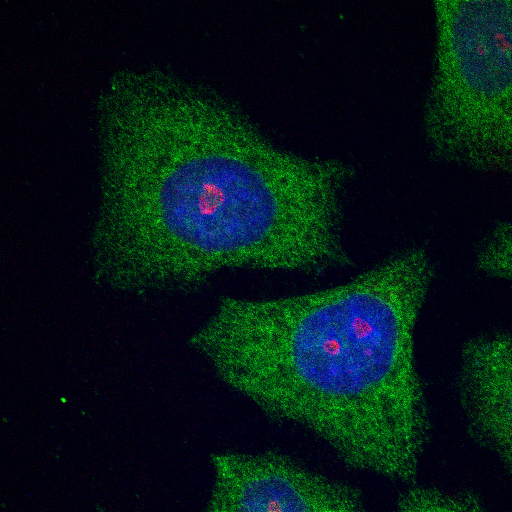

Supplement: S2 File — (ZIP) [file pone.0136964.s003.zip › S2_File/68.tif]

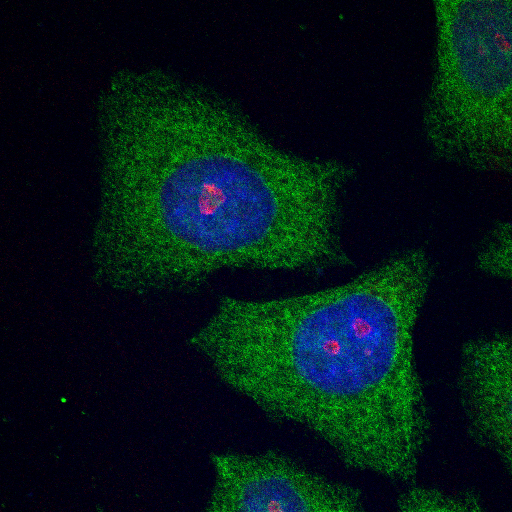

Supplement: S2 File — (ZIP) [file pone.0136964.s003.zip › S2_File/69.tif]

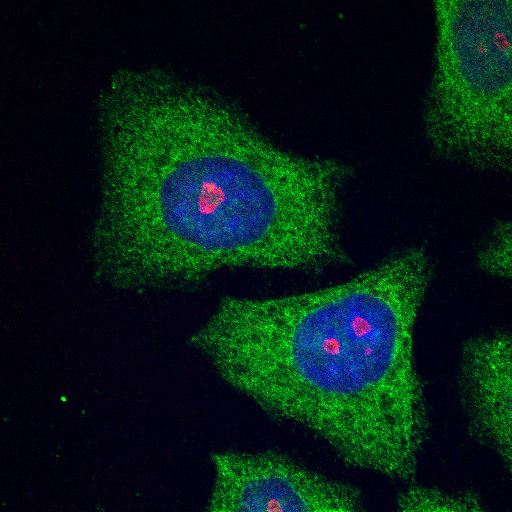

Supplement: S2 File — (ZIP) [file pone.0136964.s003.zip › S2_File/7.tif]

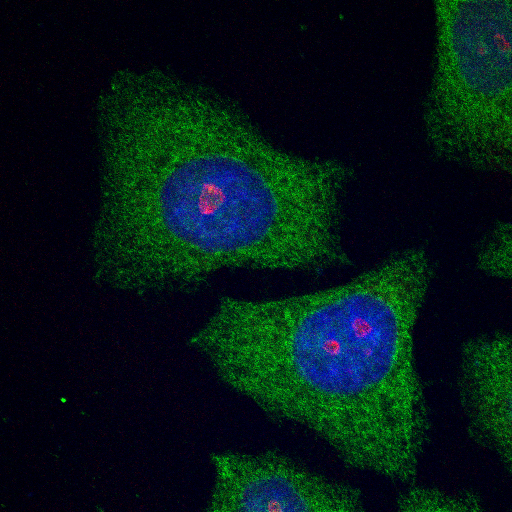

Supplement: S2 File — (ZIP) [file pone.0136964.s003.zip › S2_File/70.tif]

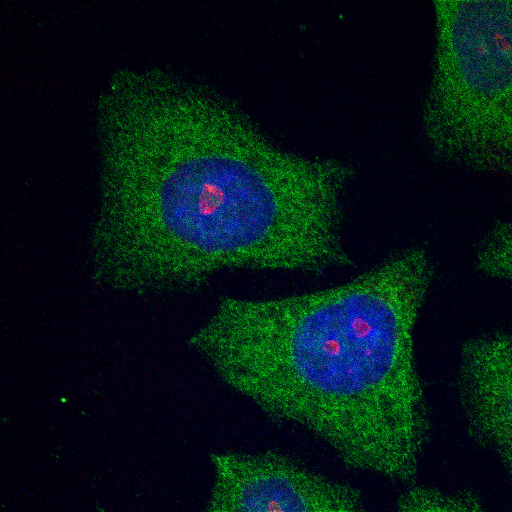

Supplement: S2 File — (ZIP) [file pone.0136964.s003.zip › S2_File/71.tif]

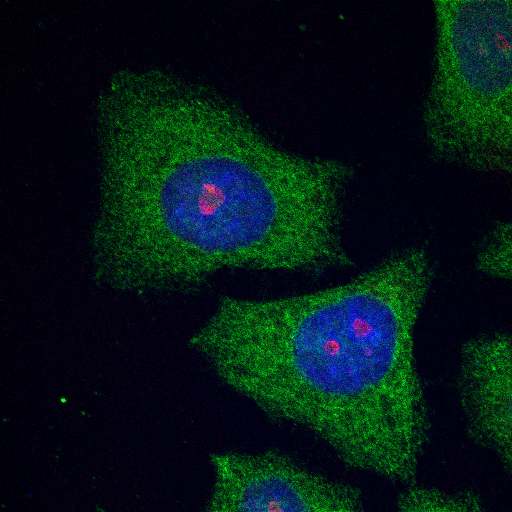

Supplement: S2 File — (ZIP) [file pone.0136964.s003.zip › S2_File/72.tif]

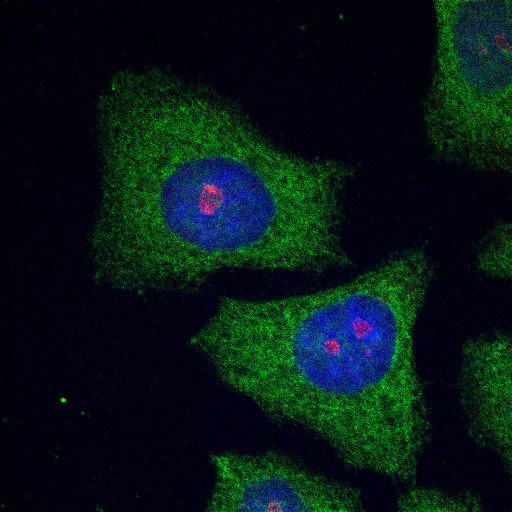

Supplement: S2 File — (ZIP) [file pone.0136964.s003.zip › S2_File/73.tif]

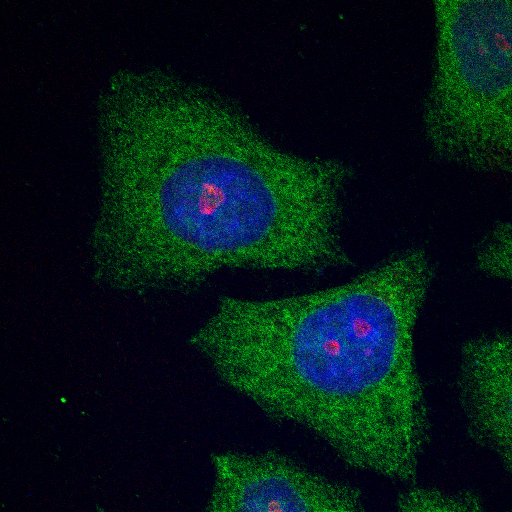

Supplement: S2 File — (ZIP) [file pone.0136964.s003.zip › S2_File/74.tif]

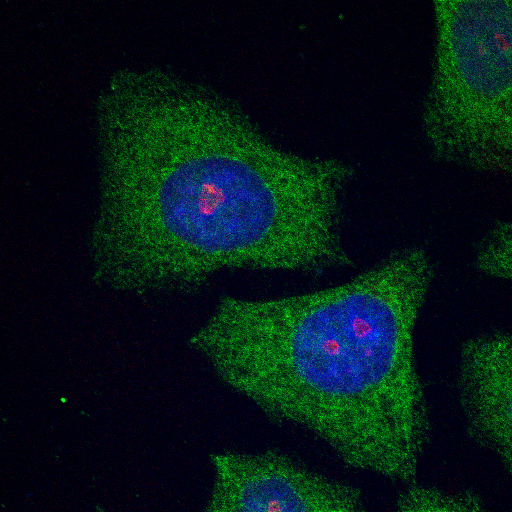

Supplement: S2 File — (ZIP) [file pone.0136964.s003.zip › S2_File/75.tif]

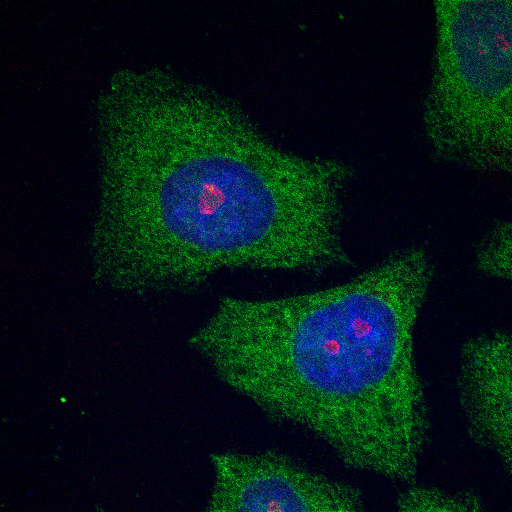

Supplement: S2 File — (ZIP) [file pone.0136964.s003.zip › S2_File/76.tif]

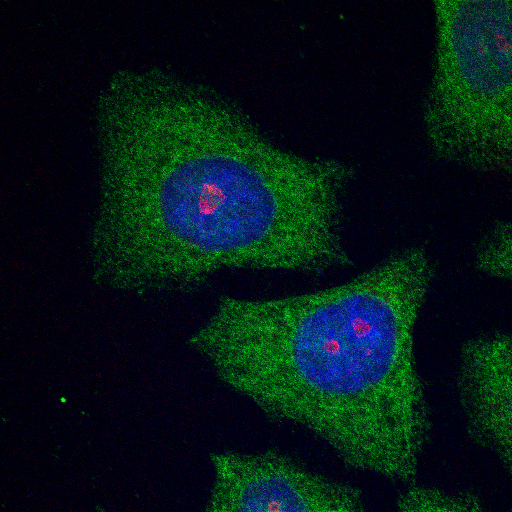

Supplement: S2 File — (ZIP) [file pone.0136964.s003.zip › S2_File/77.tif]

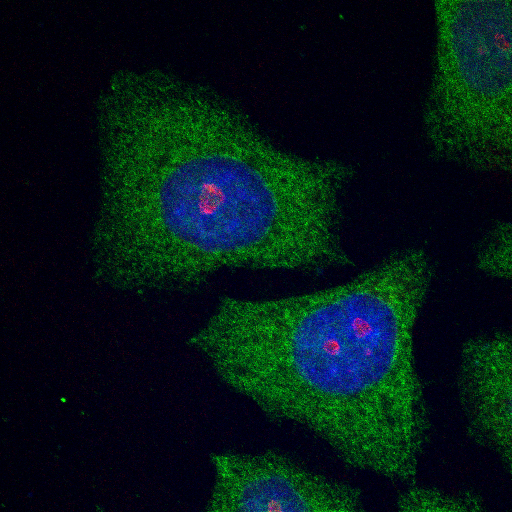

Supplement: S2 File — (ZIP) [file pone.0136964.s003.zip › S2_File/78.tif]

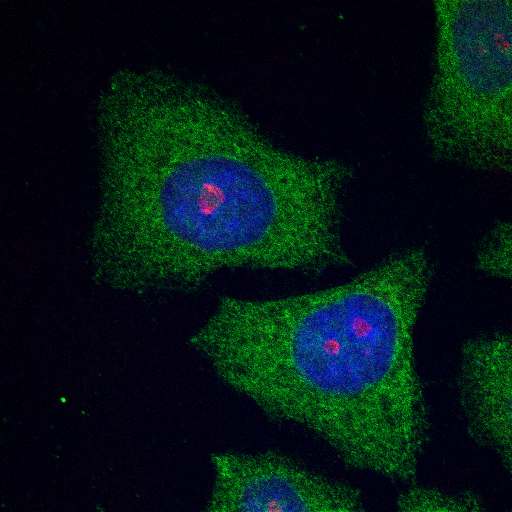

Supplement: S2 File — (ZIP) [file pone.0136964.s003.zip › S2_File/79.tif]

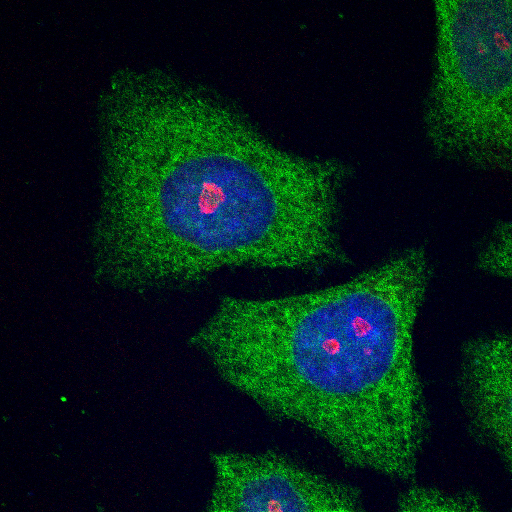

Supplement: S2 File — (ZIP) [file pone.0136964.s003.zip › S2_File/8.tif]

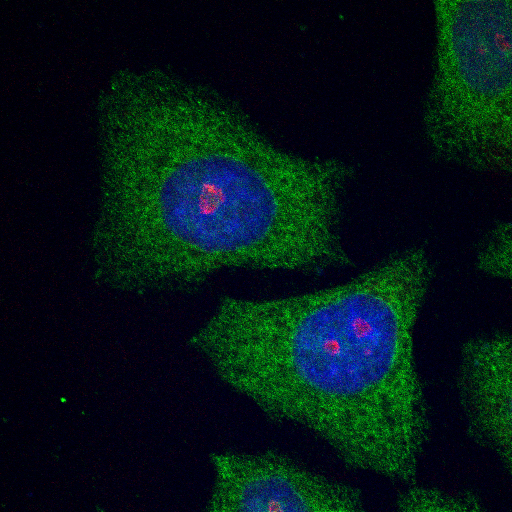

Supplement: S2 File — (ZIP) [file pone.0136964.s003.zip › S2_File/80.tif]

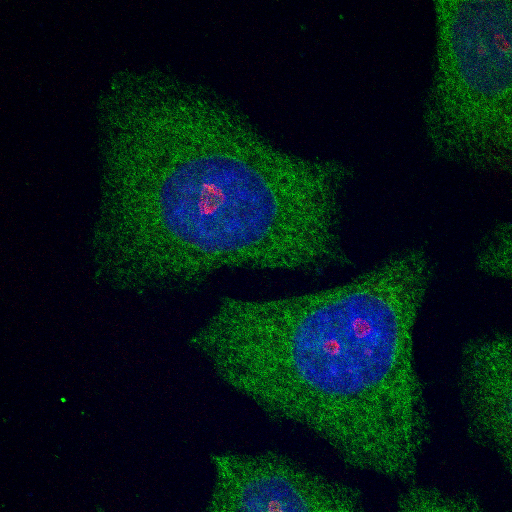

Supplement: S2 File — (ZIP) [file pone.0136964.s003.zip › S2_File/81.tif]

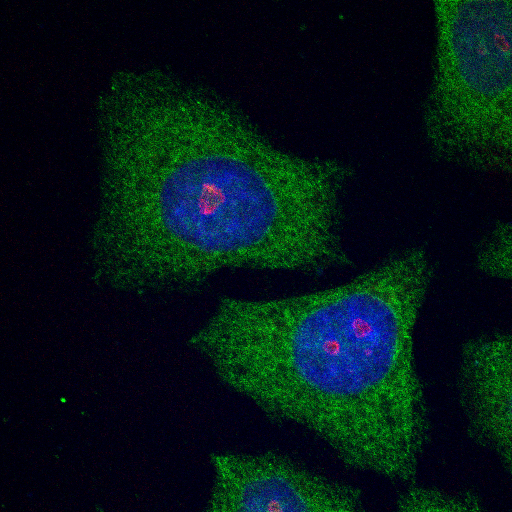

Supplement: S2 File — (ZIP) [file pone.0136964.s003.zip › S2_File/82.tif]

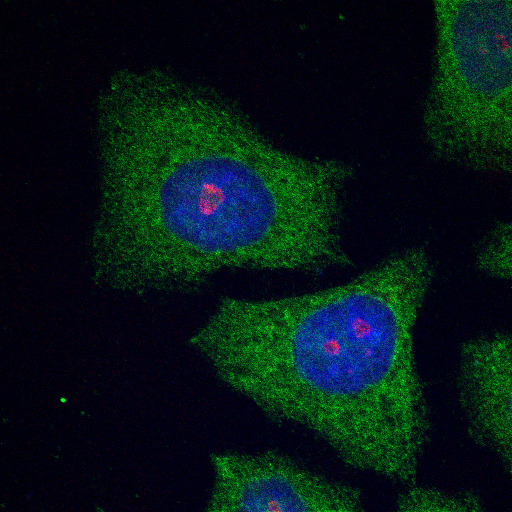

Supplement: S2 File — (ZIP) [file pone.0136964.s003.zip › S2_File/83.tif]

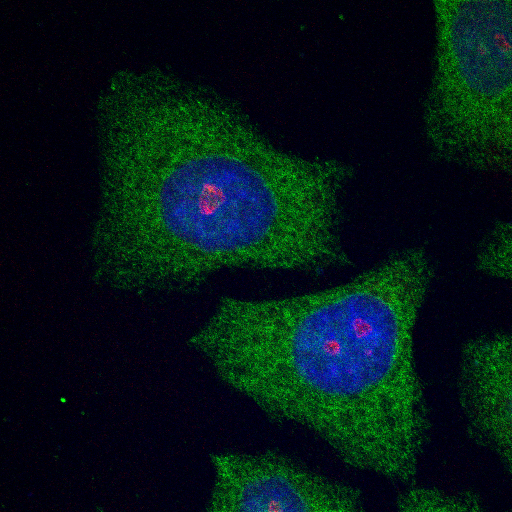

Supplement: S2 File — (ZIP) [file pone.0136964.s003.zip › S2_File/84.tif]

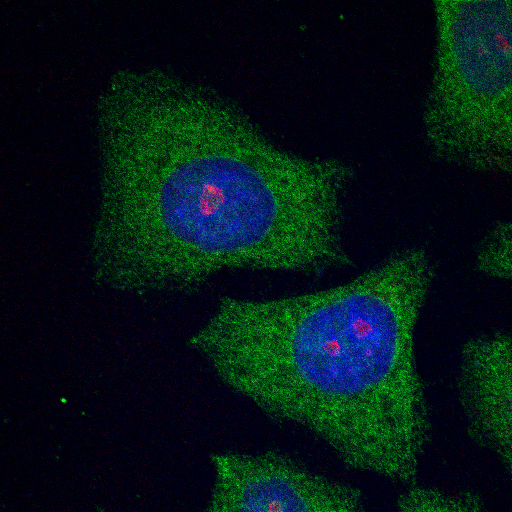

Supplement: S2 File — (ZIP) [file pone.0136964.s003.zip › S2_File/85.tif]

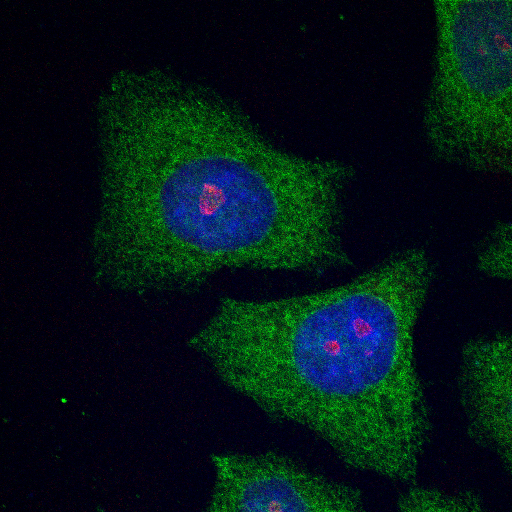

Supplement: S2 File — (ZIP) [file pone.0136964.s003.zip › S2_File/86.tif]

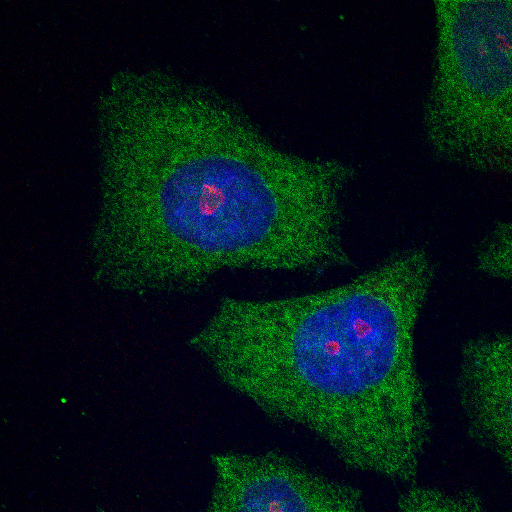

Supplement: S2 File — (ZIP) [file pone.0136964.s003.zip › S2_File/87.tif]

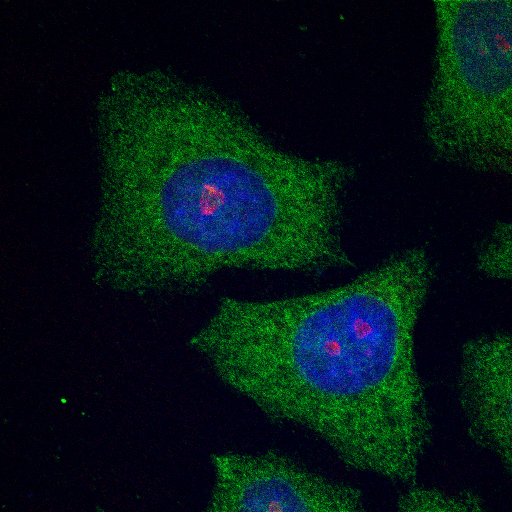

Supplement: S2 File — (ZIP) [file pone.0136964.s003.zip › S2_File/88.tif]

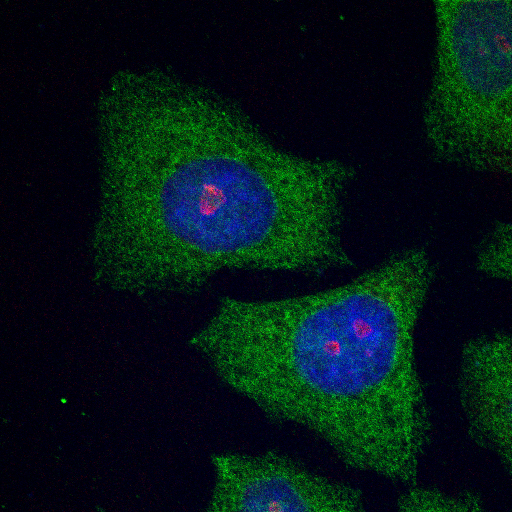

Supplement: S2 File — (ZIP) [file pone.0136964.s003.zip › S2_File/89.tif]

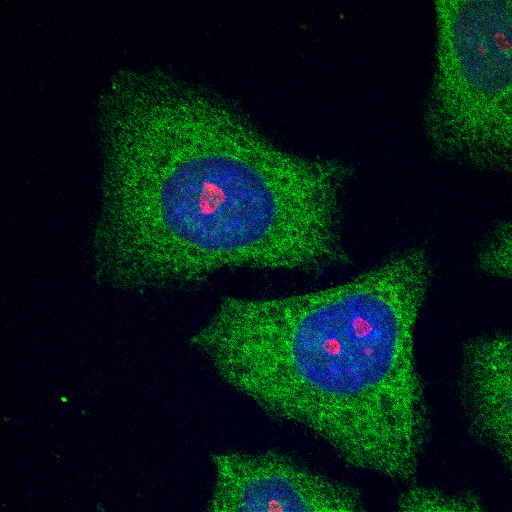

Supplement: S2 File — (ZIP) [file pone.0136964.s003.zip › S2_File/9.tif]

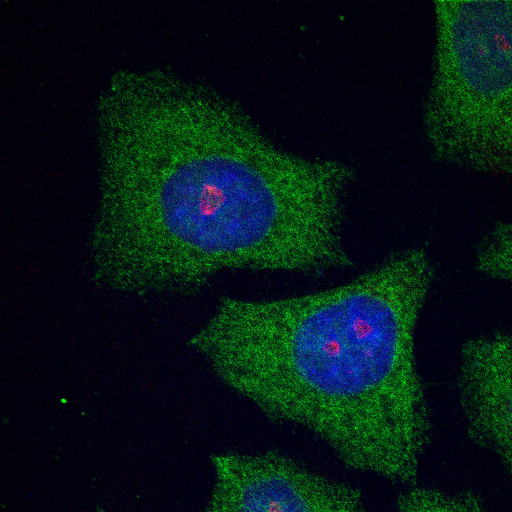

Supplement: S2 File — (ZIP) [file pone.0136964.s003.zip › S2_File/90.tif]

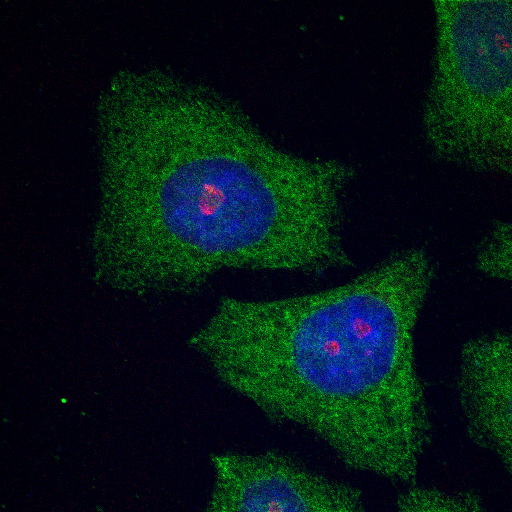

Supplement: S2 File — (ZIP) [file pone.0136964.s003.zip › S2_File/91.tif]

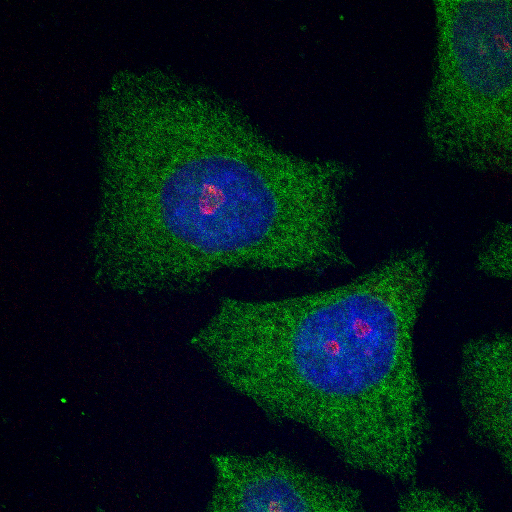

Supplement: S2 File — (ZIP) [file pone.0136964.s003.zip › S2_File/92.tif]

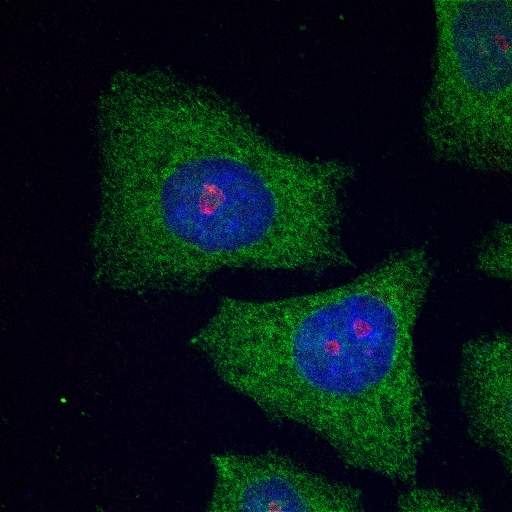

Supplement: S2 File — (ZIP) [file pone.0136964.s003.zip › S2_File/93.tif]

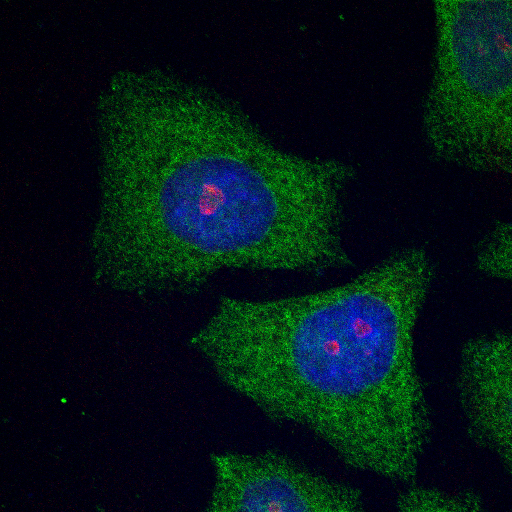

Supplement: S2 File — (ZIP) [file pone.0136964.s003.zip › S2_File/94.tif]
