# Supplementary figures and images for: Poisson-Gaussian Noise Reduction Using the Hidden Markov Model in Contourlet Domain for Fluorescence Microscopy Images (part 2 of 2)
Source: PLoS One. 2015 Sep 9;10(9):e0136964. doi: 10.1371/journal.pone.0136964 (PMC4564212; doi:10.1371/journal.pone.0136964)

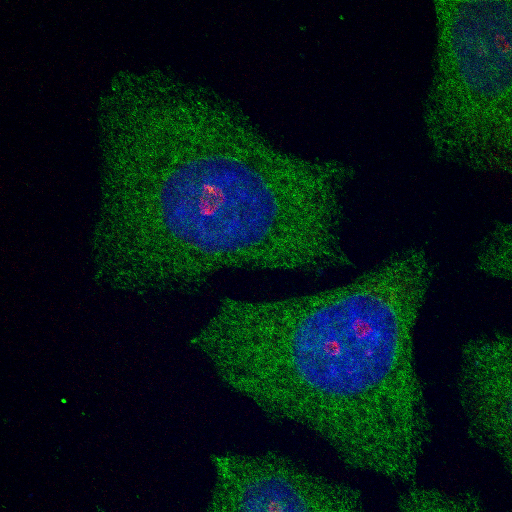

Supplement: S2 File — (ZIP) [file pone.0136964.s003.zip › S2_File/95.tif]

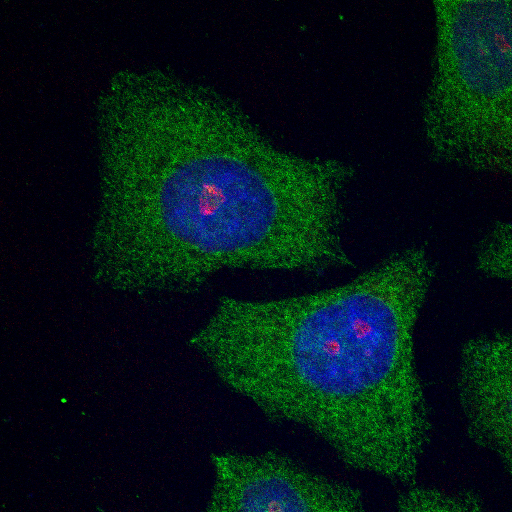

Supplement: S2 File — (ZIP) [file pone.0136964.s003.zip › S2_File/96.tif]

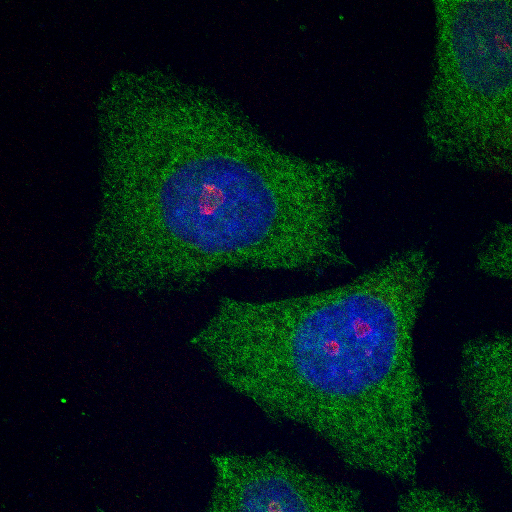

Supplement: S2 File — (ZIP) [file pone.0136964.s003.zip › S2_File/97.tif]

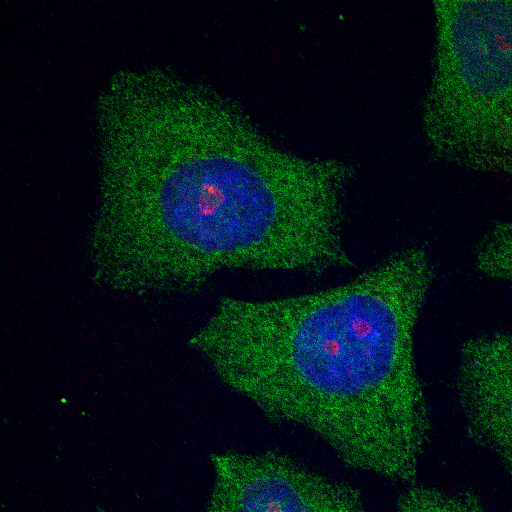

Supplement: S2 File — (ZIP) [file pone.0136964.s003.zip › S2_File/98.tif]

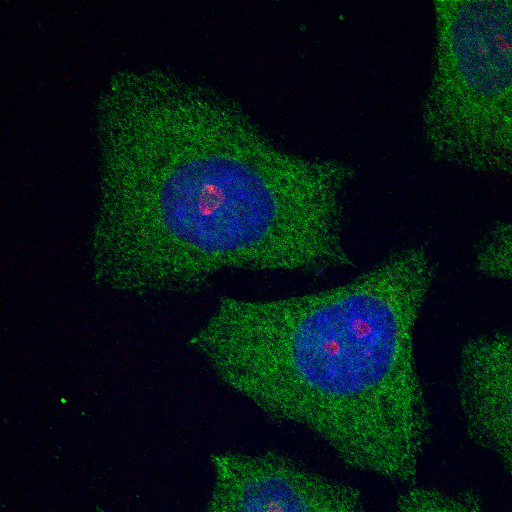

Supplement: S2 File — (ZIP) [file pone.0136964.s003.zip › S2_File/99.tif]

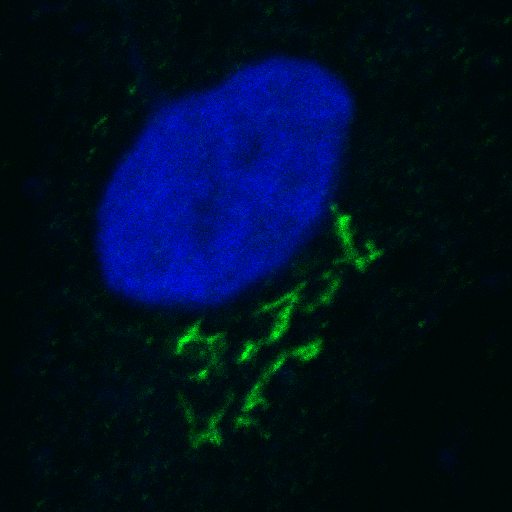

Supplement: S3 File — (ZIP) [file pone.0136964.s004.zip › S3_File/ (1).tif]

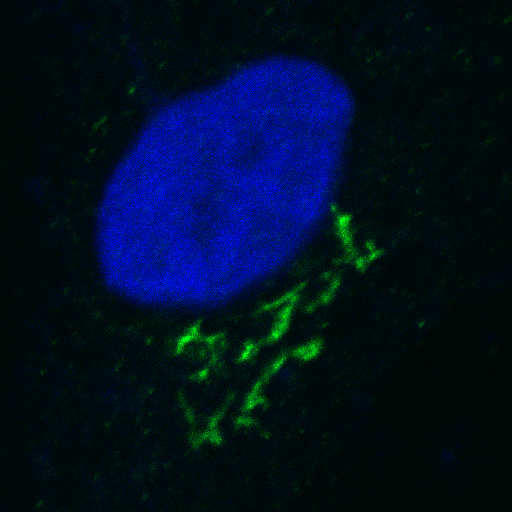

Supplement: S3 File — (ZIP) [file pone.0136964.s004.zip › S3_File/ (10).tif]

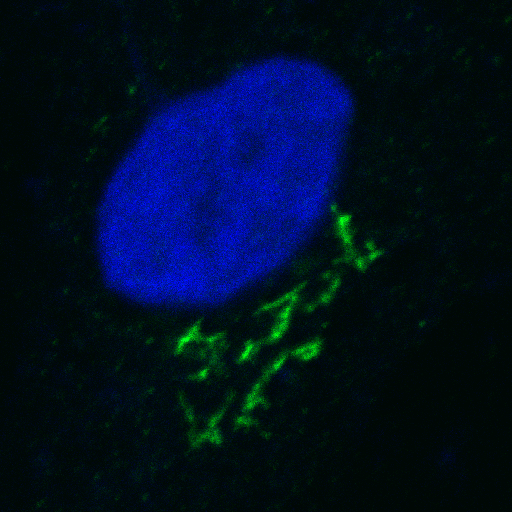

Supplement: S3 File — (ZIP) [file pone.0136964.s004.zip › S3_File/ (11).tif]

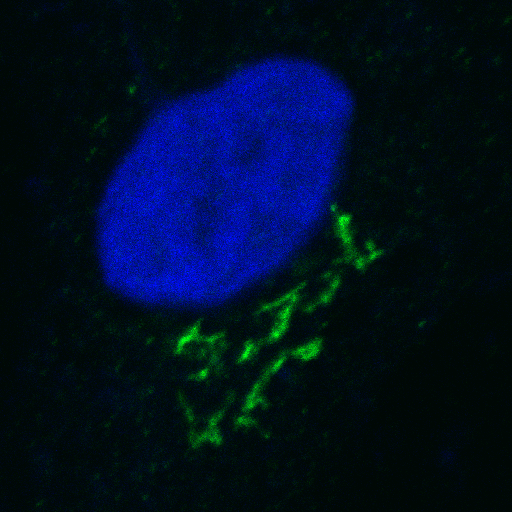

Supplement: S3 File — (ZIP) [file pone.0136964.s004.zip › S3_File/ (12).tif]

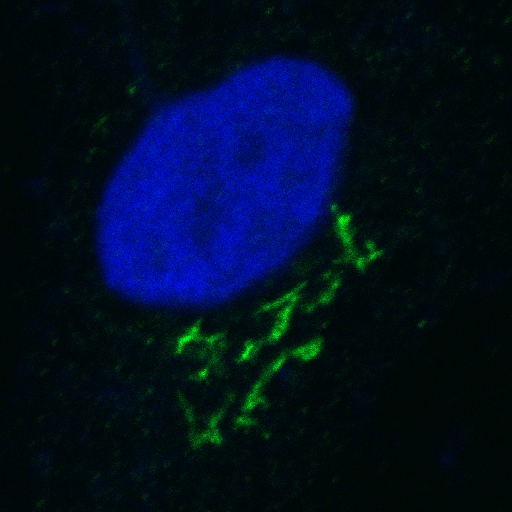

Supplement: S3 File — (ZIP) [file pone.0136964.s004.zip › S3_File/ (13).tif]

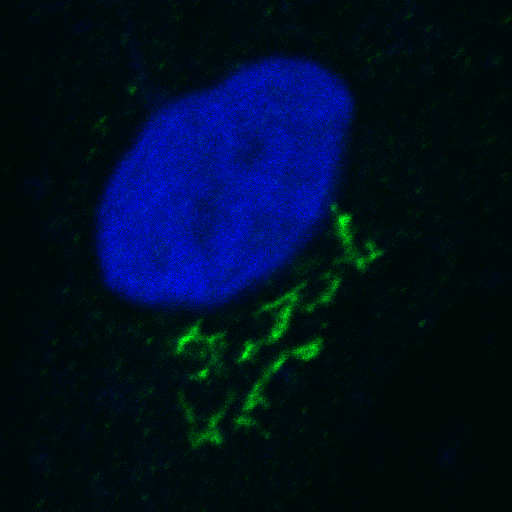

Supplement: S3 File — (ZIP) [file pone.0136964.s004.zip › S3_File/ (14).tif]

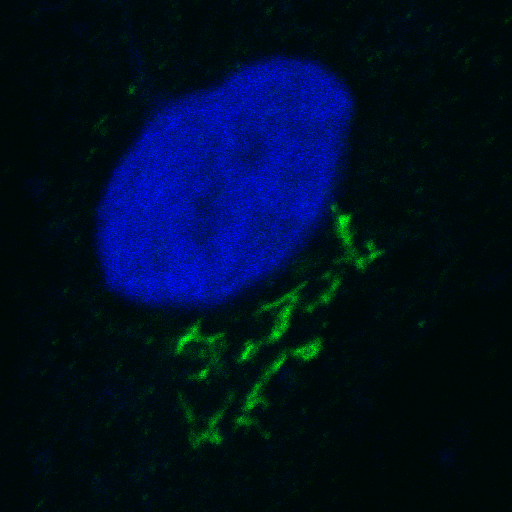

Supplement: S3 File — (ZIP) [file pone.0136964.s004.zip › S3_File/ (15).tif]

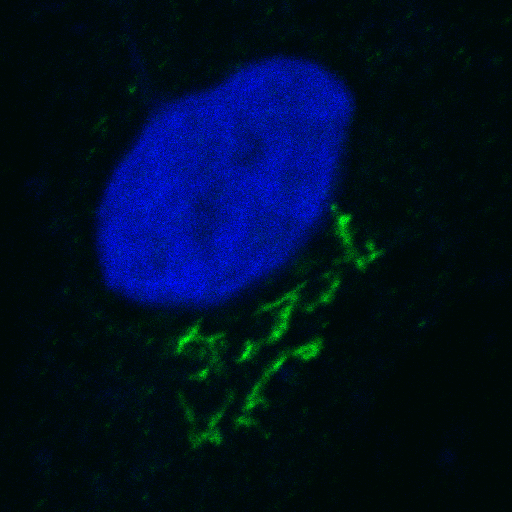

Supplement: S3 File — (ZIP) [file pone.0136964.s004.zip › S3_File/ (16).tif]

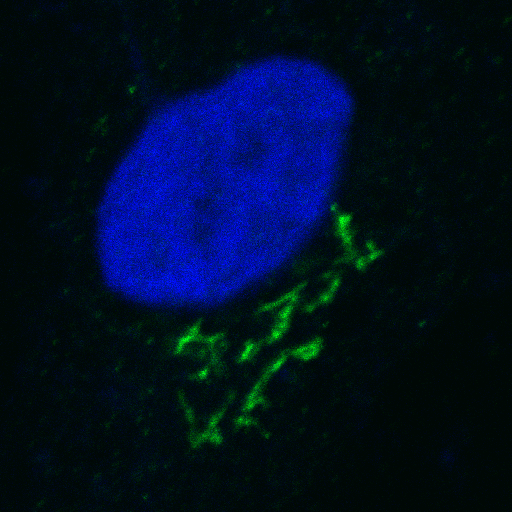

Supplement: S3 File — (ZIP) [file pone.0136964.s004.zip › S3_File/ (17).tif]

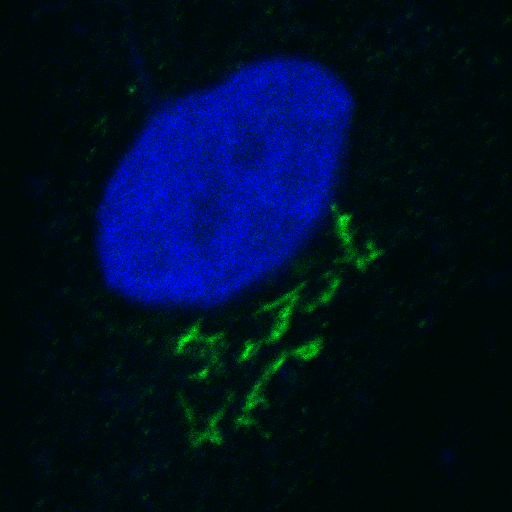

Supplement: S3 File — (ZIP) [file pone.0136964.s004.zip › S3_File/ (18).tif]

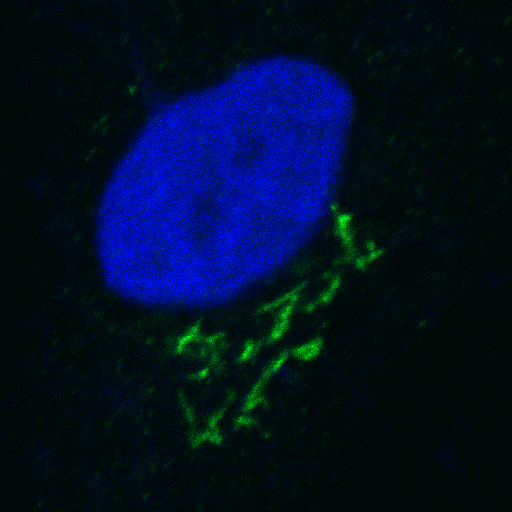

Supplement: S3 File — (ZIP) [file pone.0136964.s004.zip › S3_File/ (19).tif]

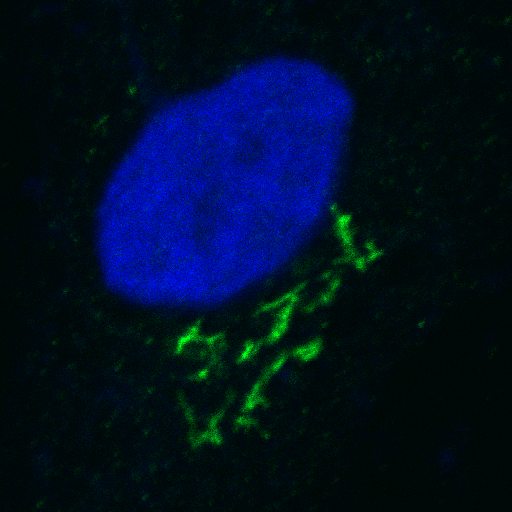

Supplement: S3 File — (ZIP) [file pone.0136964.s004.zip › S3_File/ (2).tif]

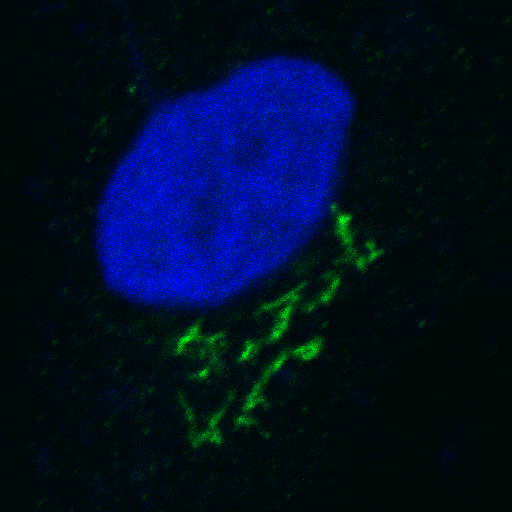

Supplement: S3 File — (ZIP) [file pone.0136964.s004.zip › S3_File/ (20).tif]

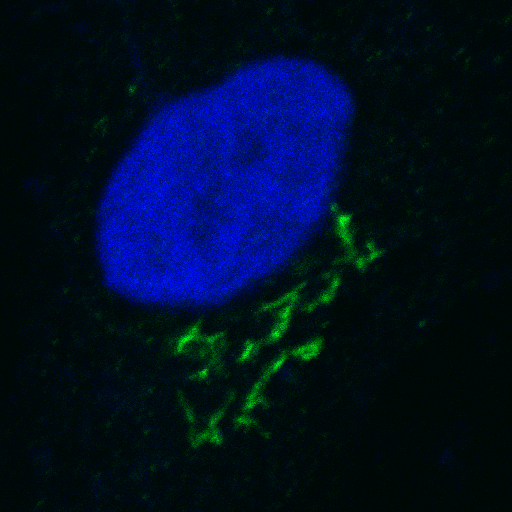

Supplement: S3 File — (ZIP) [file pone.0136964.s004.zip › S3_File/ (21).tif]

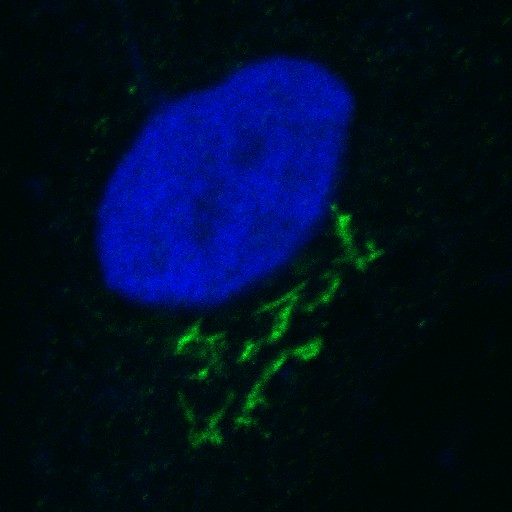

Supplement: S3 File — (ZIP) [file pone.0136964.s004.zip › S3_File/ (22).tif]

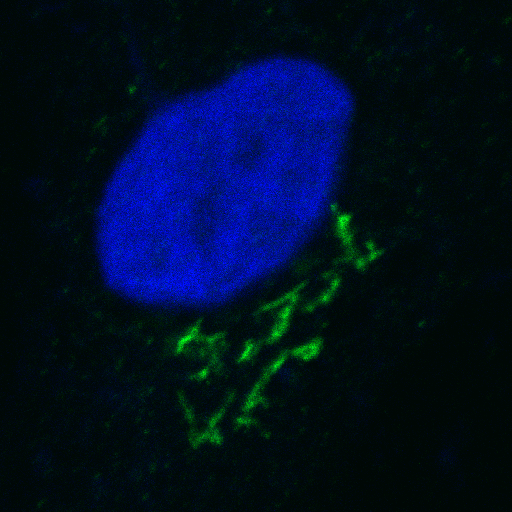

Supplement: S3 File — (ZIP) [file pone.0136964.s004.zip › S3_File/ (23).tif]

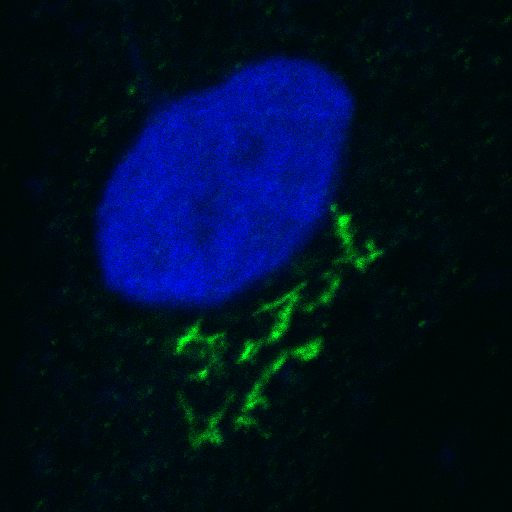

Supplement: S3 File — (ZIP) [file pone.0136964.s004.zip › S3_File/ (24).tif]

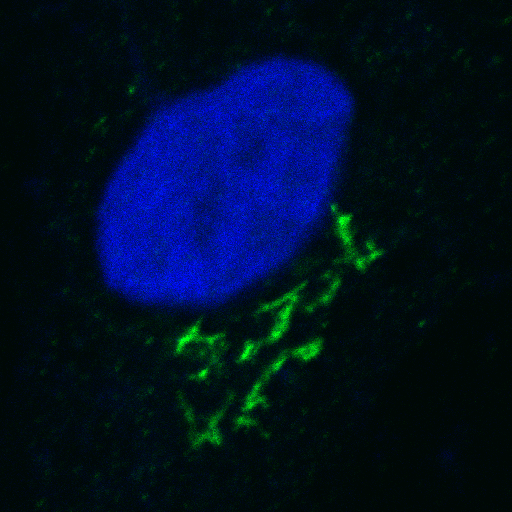

Supplement: S3 File — (ZIP) [file pone.0136964.s004.zip › S3_File/ (25).tif]

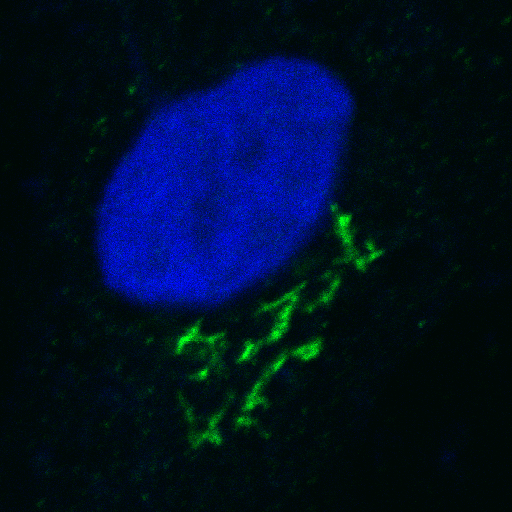

Supplement: S3 File — (ZIP) [file pone.0136964.s004.zip › S3_File/ (26).tif]

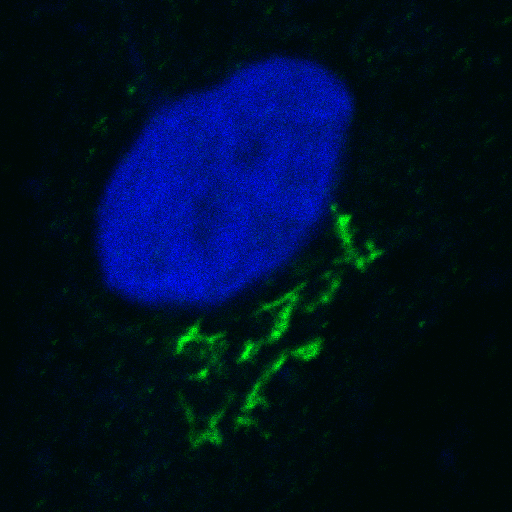

Supplement: S3 File — (ZIP) [file pone.0136964.s004.zip › S3_File/ (27).tif]

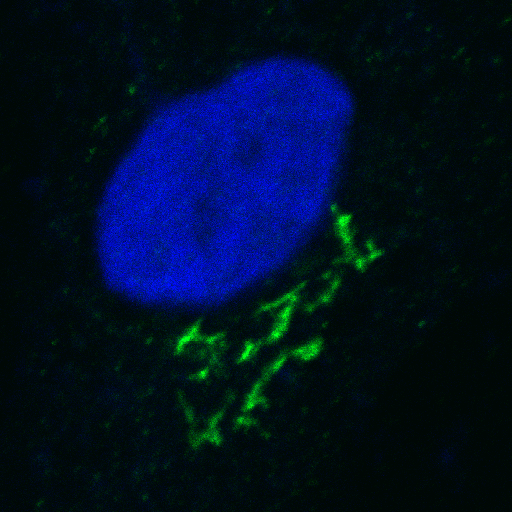

Supplement: S3 File — (ZIP) [file pone.0136964.s004.zip › S3_File/ (28).tif]

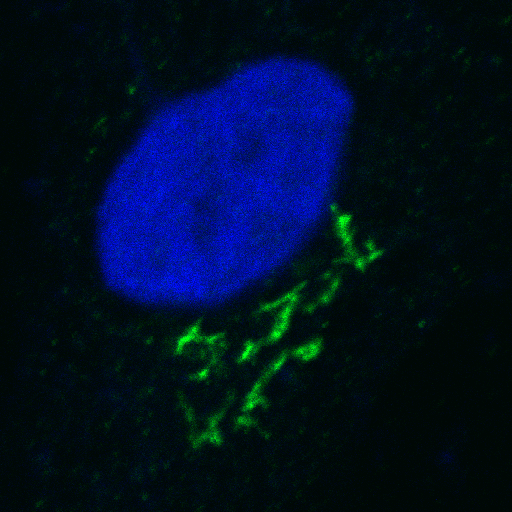

Supplement: S3 File — (ZIP) [file pone.0136964.s004.zip › S3_File/ (29).tif]

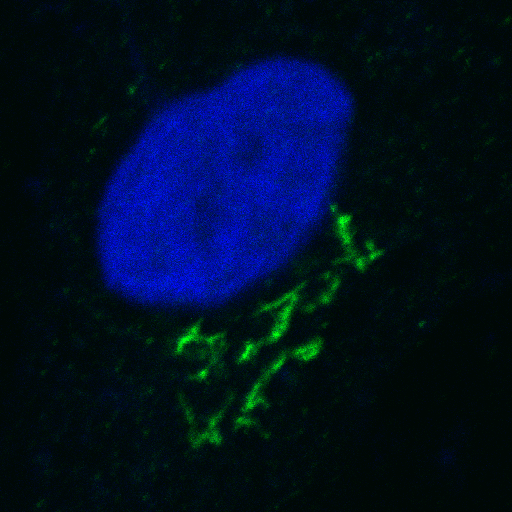

Supplement: S3 File — (ZIP) [file pone.0136964.s004.zip › S3_File/ (3).tif]

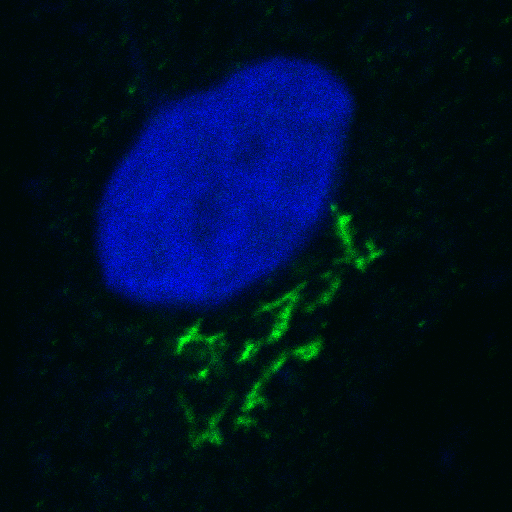

Supplement: S3 File — (ZIP) [file pone.0136964.s004.zip › S3_File/ (30).tif]

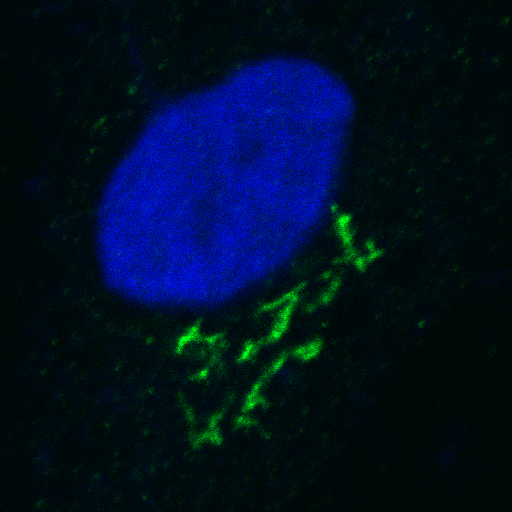

Supplement: S3 File — (ZIP) [file pone.0136964.s004.zip › S3_File/ (31).tif]

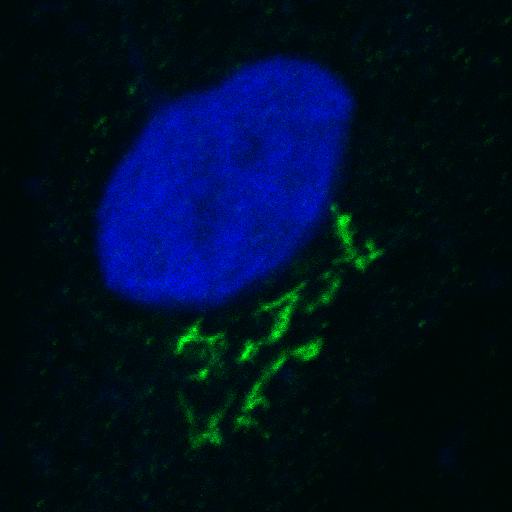

Supplement: S3 File — (ZIP) [file pone.0136964.s004.zip › S3_File/ (32).tif]

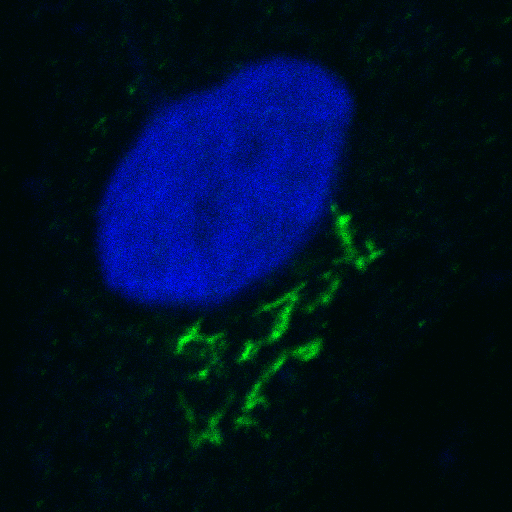

Supplement: S3 File — (ZIP) [file pone.0136964.s004.zip › S3_File/ (33).tif]

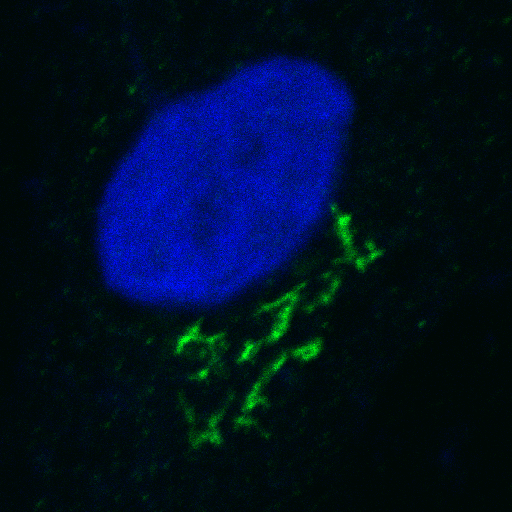

Supplement: S3 File — (ZIP) [file pone.0136964.s004.zip › S3_File/ (34).tif]

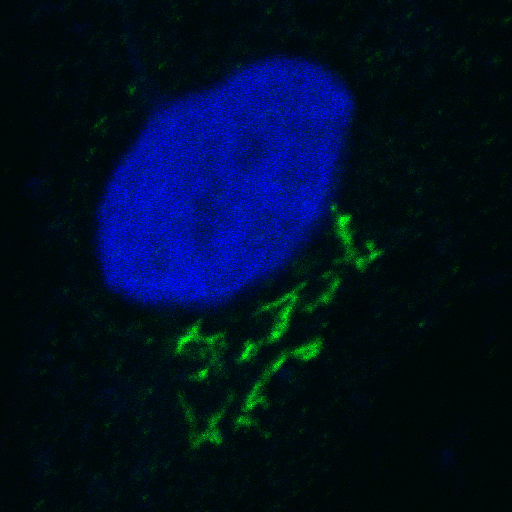

Supplement: S3 File — (ZIP) [file pone.0136964.s004.zip › S3_File/ (35).tif]

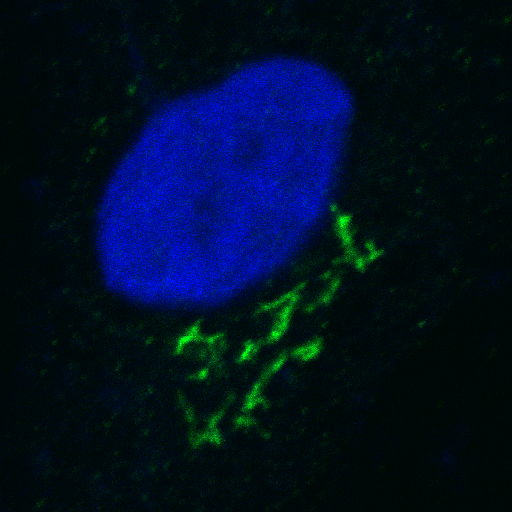

Supplement: S3 File — (ZIP) [file pone.0136964.s004.zip › S3_File/ (36).tif]

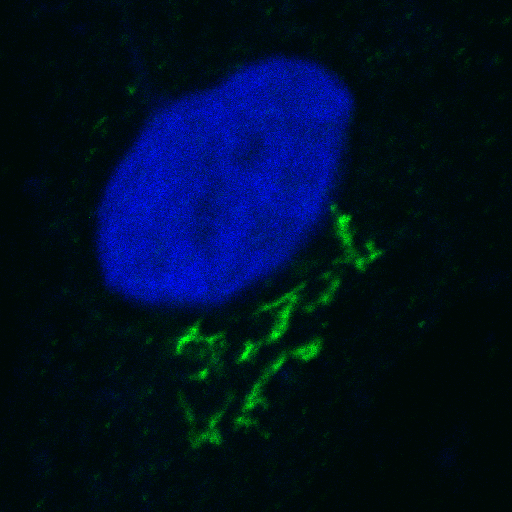

Supplement: S3 File — (ZIP) [file pone.0136964.s004.zip › S3_File/ (37).tif]

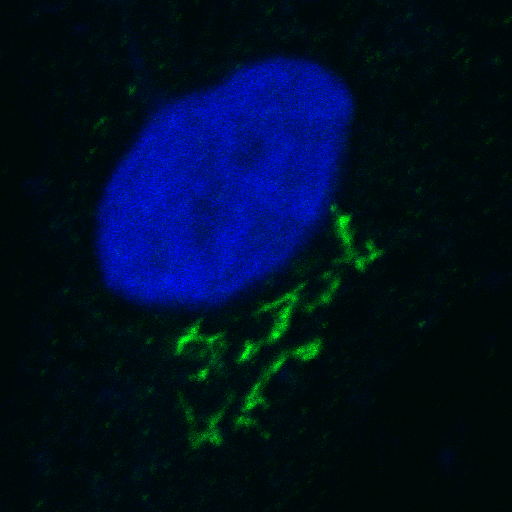

Supplement: S3 File — (ZIP) [file pone.0136964.s004.zip › S3_File/ (38).tif]

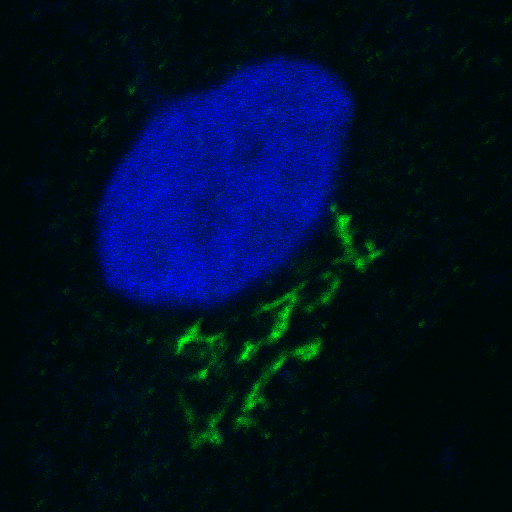

Supplement: S3 File — (ZIP) [file pone.0136964.s004.zip › S3_File/ (39).tif]

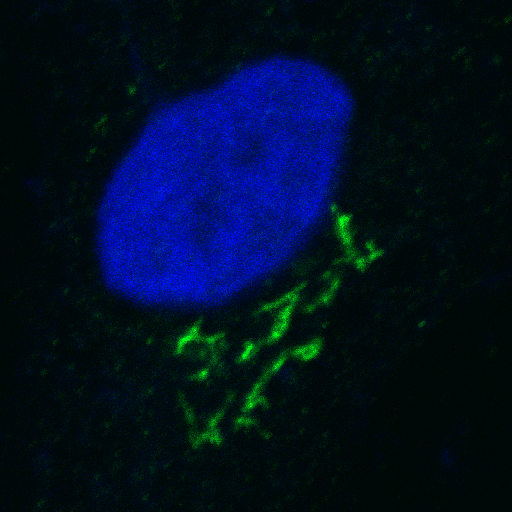

Supplement: S3 File — (ZIP) [file pone.0136964.s004.zip › S3_File/ (4).tif]

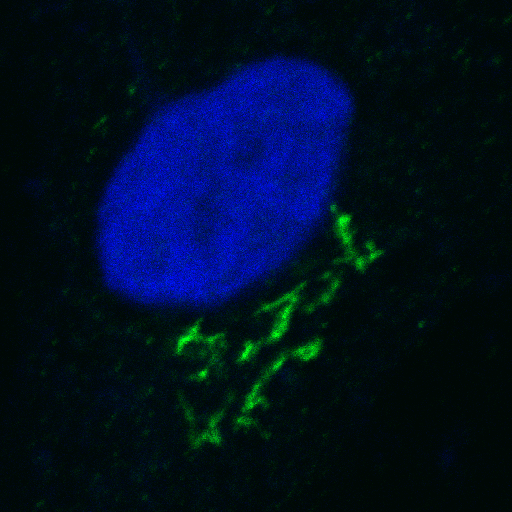

Supplement: S3 File — (ZIP) [file pone.0136964.s004.zip › S3_File/ (40).tif]

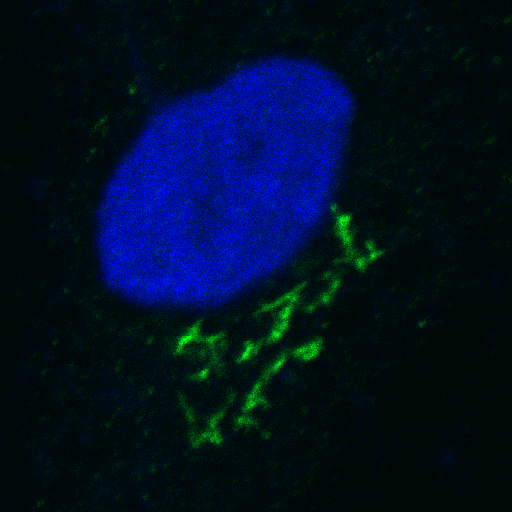

Supplement: S3 File — (ZIP) [file pone.0136964.s004.zip › S3_File/ (5).tif]

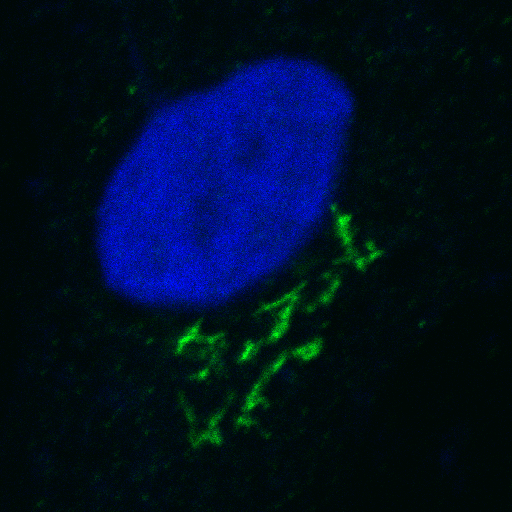

Supplement: S3 File — (ZIP) [file pone.0136964.s004.zip › S3_File/ (6).tif]

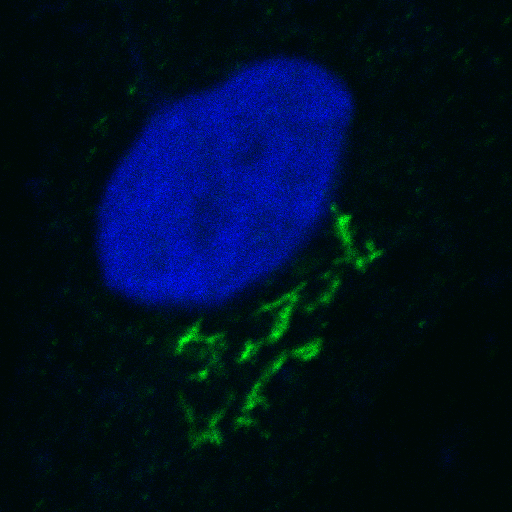

Supplement: S3 File — (ZIP) [file pone.0136964.s004.zip › S3_File/ (7).tif]

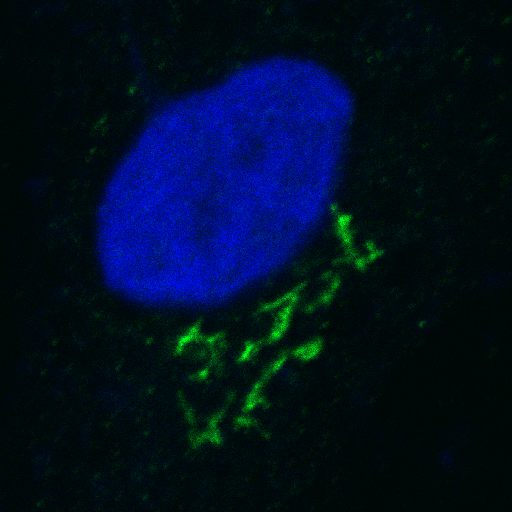

Supplement: S3 File — (ZIP) [file pone.0136964.s004.zip › S3_File/ (8).tif]

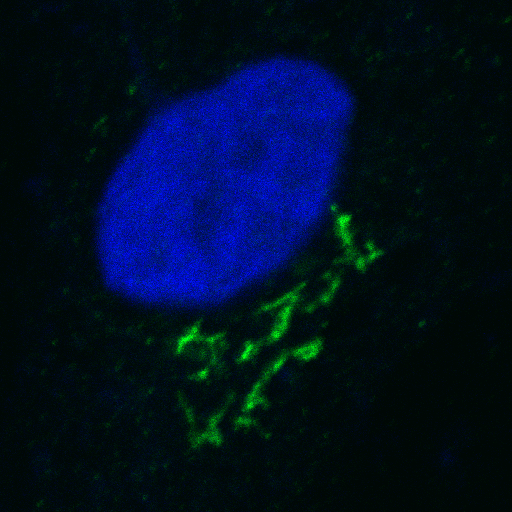

Supplement: S3 File — (ZIP) [file pone.0136964.s004.zip › S3_File/ (9).tif]
